# Systematic review on effects of bioenergy from edible versus inedible feedstocks on food security

**DOI:** 10.1038/s41538-021-00091-6

**Published:** 2021-05-04

**Authors:** Selena Ahmed, Teresa Warne, Erin Smith, Hannah Goemann, Greta Linse, Mark Greenwood, Jeremy Kedziora, Meghan Sapp, Debra Kraner, Kelli Roemer, Julia H. Haggerty, Meghann Jarchow, David Swanson, Benjamin Poulter, Paul C. Stoy

**Affiliations:** 1 Food and Health Lab, Department of Health and Human Development, Montana State University, Bozeman, MT USA; 2 Chemical and Biological Engineering Department, Montana State University, Bozeman, MT USA; 3 Statistical Consulting and Research Services, Department of Mathematical Sciences, Montana State University, Bozeman, MT USA; 4 Department of Political Science and the Department of Atmospheric Science, University of Wisconsin—Madison, Madison, WI USA; 5 Resources and Communities Research Group, Department of Earth Sciences, Montana State University, Bozeman, MT USA; 6 Sustainability Program, Department of Biology, University of South Dakota, Vermillion, SD USA; 7 Missouri River Institute, Department of Biology, University of South Dakota, Vermillion, SD USA; 8 Earth Sciences Division, NASA Goddard Space Flight Center, Greenbelt, MD USA; 9 Department of Biological Systems Engineering, University of Wisconsin—Madison, Madison, WI USA; 10 Department of Land Resources and Environmental Science, Montana State University, Bozeman, MT USA

**Keywords:** Agriculture, Sustainability

## Abstract

Achieving food security is a critical challenge of the Anthropocene that may conflict with environmental and societal goals such as increased energy access. The “fuel versus food” debate coupled with climate mitigation efforts has given rise to next-generation biofuels. Findings of this systematic review indicate just over half of the studies (56% of 224 publications) reported a negative impact of bioenergy production on food security. However, no relationship was found between bioenergy feedstocks that are edible versus inedible and food security (*P* value = 0.15). A strong relationship was found between bioenergy and type of food security parameter (*P* value < 0.001), sociodemographic index of study location (*P* value = 0.001), spatial scale (*P* value < 0.001), and temporal scale (*P* value = 0.017). Programs and policies focused on bioenergy and climate mitigation should monitor multiple food security parameters at various scales over the long term toward achieving diverse sustainability goals.

## Introduction

Providing adequate nutritious food to support a growing population while conserving natural resources is a key aspect of the United Nations Sustainable Development Goals^1–3^. Many people face food insecurity, a lack of adequate access to sufficient, safe, and nutritious food that meets their dietary needs and food preferences for an active and healthy life^4–6^. Diet-related health issues are a primary risk factor of disease globally^7^. Currently, the production of crops and livestock places greater land-use stress on ecosystems compared to all other land-use activities^1,2,8,9^. Maintaining the integrity of food supply amongst a range of competing ecosystem services is becoming more critical as the population increases^10^. For example, habitat loss from the conversion of natural lands for the production of food^11,12^ and first-generation biofuels is the leading threat to biodiversity^13^. These food system challenges are exacerbated by climate change, the industrialization of agriculture, and land degradation with notable implications for human and planetary health^14–17^.

Food security is a multidimensional goal that includes parameters of food availability, food prices, and food production. Although food security goals are prioritized by international organizations and national governments, they are increasingly competing with other societal and planetary goals^18–20^. For example, there is increased societal demand for bioenergy as well as a recognized need to support ecosystem health through efforts such as afforestation for biodiversity conservation and carbon dioxide removal toward ameliorating the impacts of climate change^18–21^. A shift in priority to renewable energy globally has driven an increase in bioenergy production over the past two decades in the search of alternatives to fossil fuels^22–24^. In recognition of negative trade-offs between bioenergy (interchangeably referred to as biofuels) and food security, feedstocks from second- and third-generation biofuels (next-generation biofuels), along with newer technologies, are being developed with the goal to avoid competing with lands used for food production while simultaneously lowering greenhouse gas (GHG) emissions^23,25,26^.

The expansion of bioenergy production in the context of climate change mitigation represents a novel and poorly understood threat to food security. Impacts of bioenergy production on food security have been found to vary based on geographic location, national infrastructure, technology, and policy, global market, and a class of biofuel feedstock^27–32^. While multiple studies have reviewed the effects of bioenergy expansion on food security^22,23,33–35^, none has synthesized the totality of the evidence regarding how various bioenergy feedstocks impact different aspects of food security. This study addresses this knowledge gap through a systematic literature review that examines the following question: what are the effects of various classes of bioenergy feedstocks on food security parameters (food availability, food prices, and food production)? Bioenergy feedstocks can be variously classified; given our emphasis on food security, we classified and compared bioenergy feedstocks on the basis of being edible versus inedible for human and livestock consumption (see Box 1: Definitions and Background for further details). We further examine how this relationship varies on the basis of the sociodemographic index, spatial and temporal scale, and data type.

Box 1 Defining the different types of bioenergy feedstocks

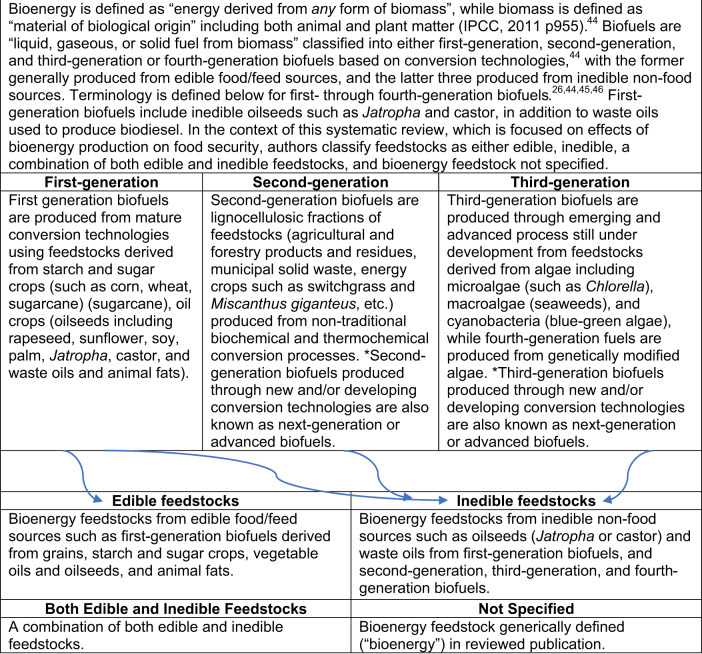

## Background

Despite a doubling of food production over the last three decades and a decline in world hunger between 2005 and 2014, trends from 2014 through 2019 highlight a rise in world hunger with an estimated 690 million people being undernourished^36^. The prevalence of severe food insecurity has increased during 2014–2019 in all regions of the world except Northern America and Europe, while Africa, Asia, Latin America, and the Caribbean are disproportionately affected^36^. A lack of food security contributes to malnutrition in the forms of obesity and undernutrition and their co-existence. The recent rise in global hunger and food insecurity has been attributed to climate variability and extremes, land-use change, conflict, and economic fluctuations^20^. Supporting food security goals thus must consider climate mitigation and adaptation in a world where atmospheric concentrations of greenhouse gases continue to increase as a result of anthropogenic activities^37,38^.

While efforts to support food security contribute to achieving multiple UN Sustainable Development Goals (SDGs), including ending hunger (SDG 2) and ensuring healthy lives (SDG 3), they may conflict with other SDGs, including increased energy access (SDG 7) and combatting climate change and its impacts (SDG 13)^39^. In line with SDG 7 and 13, bioenergy has expanded^23^ to account for ~10% of global energy and 70% of renewable energy^24^. The production of bioenergy has various consequences for food security at the household, community, regional, and national levels^18,22,40,41^. For example, bioenergy production can impact food security by competing for land, labor, water, and other resources, and often decrease food availability while increasing food prices, though effects are variable based on bioenergy feedstock^18,40^. In contrast to first-generation biofuels produced on arable lands, next-generation biofuels produced on marginal lands not used for food production have emerged to address the negative implications of first-generation biofuels on food security by avoiding competition with food production, while having relatively lower GHG emissions^23,25,26,42,43^.

However, the impacts of bioenergy on food security are not always clear on the basis of the ways biofuel feedstocks are produced and given that there are multiple dimensions of food security. Bioenergy may be produced on arable land competing with food production, arable land not competing with food production, or marginal and/or degraded land (that with either temporary or permanent diminished productive capacity^44^). In order to better understand the effects of different types of bioenergy, various classification schemes have been proposed. The Intergovernmental Panel on Climate Change (IPCC) recognizes three classes of biofuels that include first-generation, second-generation, and third-generation biofuels, with the latter two sometimes referred to as next-generation or advanced biofuels^44^. First-generation biofuels are created through mature conversion technologies and comprise of feedstocks from oilseeds, grains, animal fats, and waste vegetable oils (Box 1: Definitions^26,44–46^). Second-generation biofuels are created through thermochemical and biochemical conversion technologies from lignocellulosic biomass, such as agricultural and forest residues, perennial herbaceous plants, and short-rotation woody crops. Third-generation biofuels are produced through various technologies such as conversion technologies that transform algal biomass feedstock.

However, while we recognize the IPCC classification of biofuels, there is a lack of clarity in the IPCC classification on the basis of their edible properties of feedstocks for human consumption. For example, first-generation biofuels may be produced from crops that are both edible and inedible for human or livestock consumption. Given the need to understand the effects of bioenergy on food security, we contend that there is a need to classify bioenergy feedstocks as edible or non-edible for human and livestock consumption. Thus, we created and implemented a new classification scheme in this systematic review for categorizing feedstocks for bioenergy production as edible or inedible (see Box 1: Definitions). We classify feedstocks as edible if they are consumed by humans or livestock as either traditional food or feed crop. Further, we classify feedstocks as inedible if they are not consumed by humans or livestock as a traditional food or feed crop. We applied this classification scheme to characterize studies included in this systematic review and address the research question.

## Results

A total of 224 publications was identified in the systematic review that met the a priori inclusion criteria to address the study question: what are the effects of various classes of bioenergy feedstocks on food security parameters (food availability, food prices, and food production)? Here, we first synthesize the number of studies based on types of bioenergy feedstocks and food security parameters examined before synthesizing the effects of bioenergy production on overall food security and specific food security parameters (food availability, food prices, food production, and multiple food security parameters). Next, we present a synthesis of the findings on the basis of various scales characterizing the studies, including sociodemographic index level, spatial scale, and temporal scale. We conclude with the type of data examined (observed versus modeled studies). Findings are reported for bioenergy effects (negative, positive, both negative and positive, and no effect) on the basis of all bioenergy feedstocks as well as on the basis of the edibility of the feedstock as edible, inedible, or both edible and inedible feedstocks examined.

### Bioenergy feedstocks

**Fig. 1:**
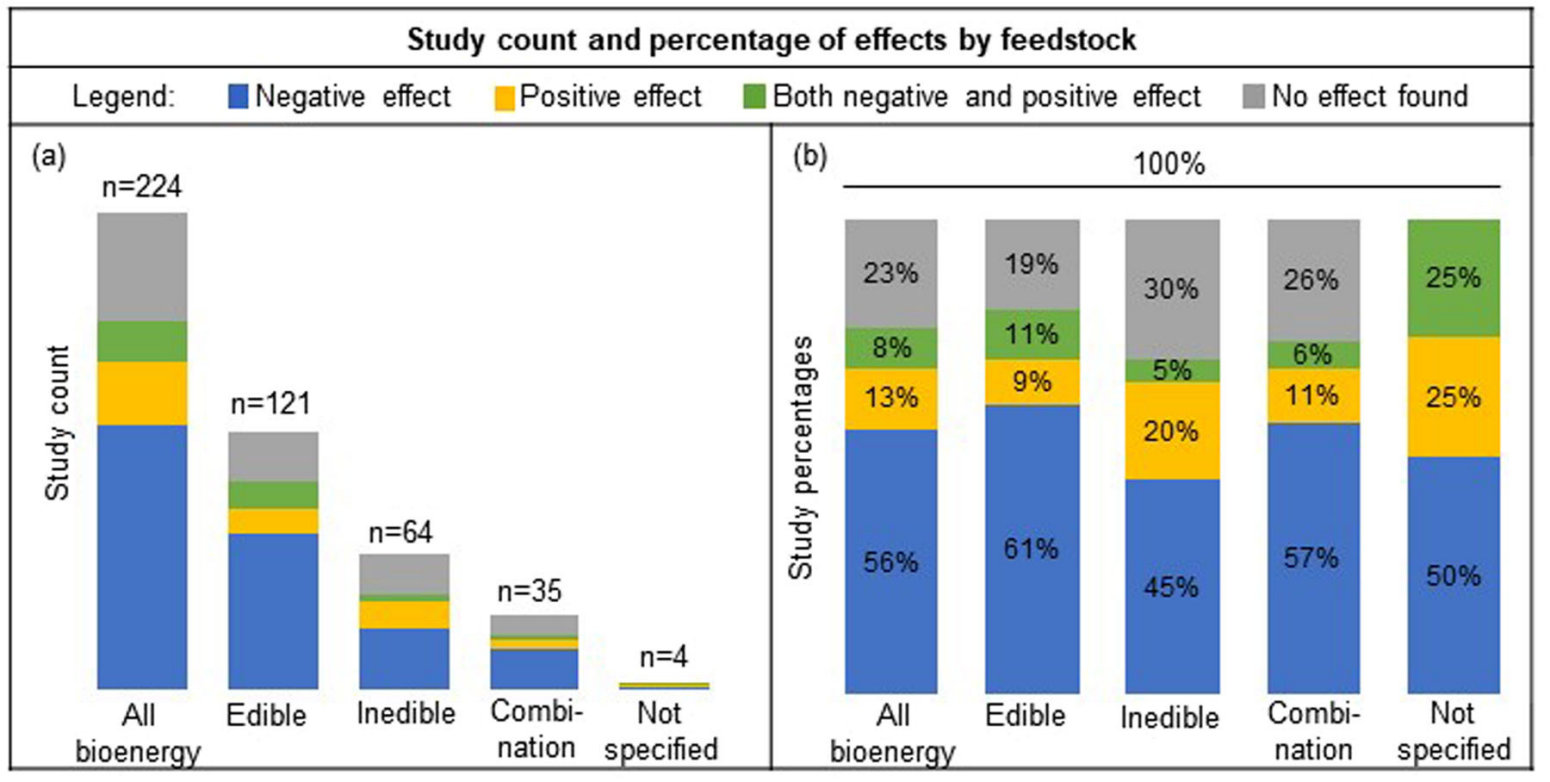
Publication count and percentage of effects by bioenergy feedstock. The count (**a**) and percentage (**b**) of publications that reported a negative, positive, combination of both negative and positive, and neutral effect of bioenergy expansion on overall food security with all bioenergy, edible, inedible, a combination of both edible and inedible bioenergy, and bioenergy type not specified.

Approximately half of the reviewed publications examined edible feedstocks (121 of 224 publications) and about one-third examined inedible feedstocks (64 publications). Fewer studies examined both edible and inedible feedstocks (35 publications) and a few studies did not specify the bioenergy feedstock examined (4 publications) (Fig. 1). Edible feedstocks examined in the studies comprised sugar and starch crops such as corn, sugarcane, sugar beet, and cassava for ethanol as well as oilseeds such as soy, canola, and palm for biodiesel. Inedible feedstocks included oilseeds such as *Jatropha* and castor, perennial switchgrass, fast-growing trees, by-products, agricultural wastes and residues, and algae. Numerous studies examined multiple feedstocks, whether only edible feedstocks (such as soy and corn), inedible feedstocks (*Miscanthus giganteus* and corn stover), or a combination of edible and inedible feedstock (such as corn and corn stover).

### Food-security parameters

Food production (29% of the 224 publications) and food price (27%) were the most prevalent food security parameters in the studies included in this systematic review (Supplementary Table 1). Measurement of food production frequently included sub-parameters such as crop displacement and land-use change, while measurement of food prices included sub-parameters of absolute price and price volatility (Supplementary Table 1). Food availability was the least prevalent food security parameter examined (12%) and most often included the sub-parameter of food insecurity of households or communities. Approximately one-third of the publications (32%) investigated multiple food security parameters.

### Relationship of bioenergy and food security

**Fig. 2:**
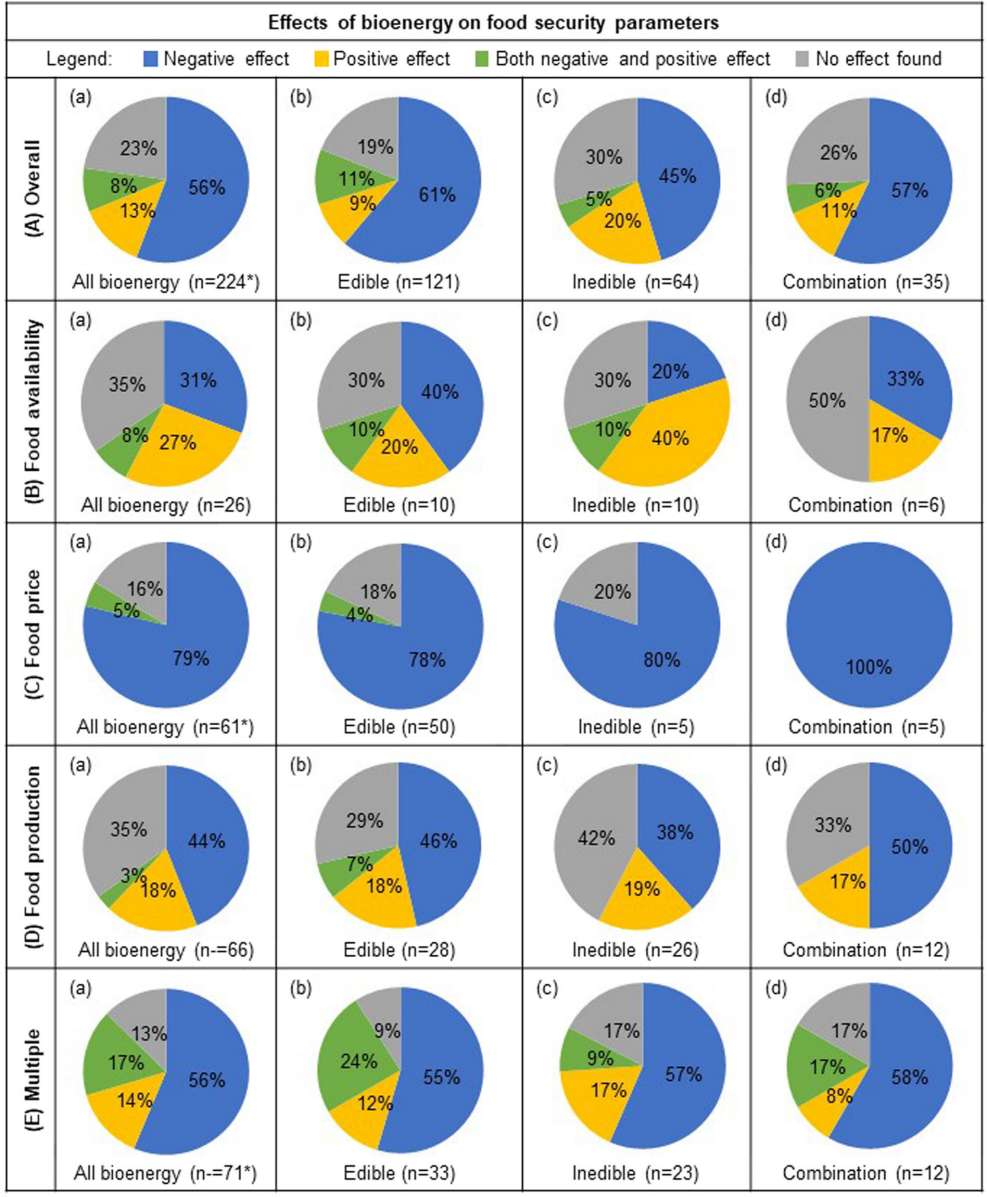
Effects of bioenergy on overall food security and food security parameters food availability, food price, food production, and multiple food security parameters. The percentage of reviewed publications (*n* = 224) that reported either negative, positive, both negative and positive, or neutral effect of all bioenergy (column **a**), edible (**b**), inedible (**c**), and studies that included a combination of both edible and inedible feedstocks in their analysis (**d**), on overall food security (row **A**), and food security parameters food availability (**B**), food price (**C**), food production (**D**), and multiple food security parameters (**E**). *Bioenergy type “not specified” (*n* = 4) is only included in the tabulation of “All bioenergy” (column **a**), and therefore the sum of edible, inedible, and combination of both edible and inedible feedstocks (columns b–d) will not necessarily be a composite of “All bioenergy”.

Just over half the publications (56% of 224 publications) reported a negative impact of bioenergy production on overall food security (overall food security includes all food security parameters examined) and approximately a quarter of the studies reported no effect (23%). Only several studies reported a positive effect of bioenergy on overall food security (13%) and a few reported both negative and positive effects (8%) (Fig. 2A: a). Further, across each of the different types of feedstocks classified on the basis of edibility (edible, inedible, and both edible and inedible feedstocks), the reported effects on overall food security were largely negative (45–61%) (Fig. 2A: b–d). Studies that reported no effect of bioenergy on food security were less prevalent across the different feedstocks classified based on edibility (19–30%), followed by positive effects (9–20%), and both negative and positive effects (5–11%). While the percentage of studies reporting negative versus positive effects of bioenergy on food security varied on the basis of the type of bioenergy feedstock (edible, inedible, or both edible and inedible), both the parametric and nonparametric chi-squared tests indicated little evidence of a relationship between the type of bioenergy feedstock (edible, inedible, or both edible and inedible) and food security (*χ*^2^ = 13. 1, permutation-based *P* value = 0.15). In contrast to type of bioenergy feedstock (edible, inedible, or both edible and inedible), both the parametric and nonparametric chi-squared tests indicated strong evidence of a relationship between bioenergy and specific food security parameters (food availability, food price, food production, and multiple food security parameters) (*χ*^2^ = 44.8, permutation-based *P* value < 0.001).

**Fig. 3:**
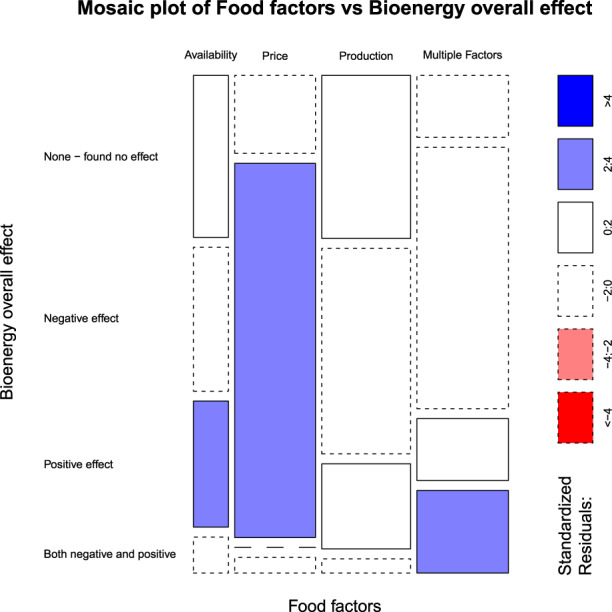
Mosaic plot of standardized residuals for the food security parameters by the overall effect. Cells that are shaded light blue indicate there are three standardized residuals that are between two and four which is larger than would be expected if there was no relationship between food security parameters and overall effect. Cells with solid borders indicate positive residuals, whereas cells with dashed borders indicate negative residuals.

For the food security parameter of food availability, approximately one-third of the articles reported no effect of all types of bioenergy (35% of 26 publications), approximately another third reported negative effects (31%), and just under a third reported positive effects (27%) (Fig. 2B). A few publications reported both a negative and positive effect of bioenergy on food availability (8%) (Fig. 2B). For the food security parameter of food price, over three-quarters of the publications reported a negative effect of bioenergy production on food price (79% of 61 publications) and the remaining studies reported no effect (16%) or both negative and positive effects (5%) (Fig. 2C). None of the publications reported solely positive effects of bioenergy production on food prices. For the food security parameter of food production, almost half the publications reported negative effects of bioenergy production on food production (44% of 66 publications) and approximately one-third of studies reported no effect (35%) (Fig. 2D). Several studies reported positive effects of bioenergy on food production (18%) and a few reported both negative and positive effects (3%) (Fig. 2D). Over half the publications investigating the effect of bioenergy on multiple food security parameters reported negative effects (56% of 71 publications), while several publications reported both negative and positive effects (17%), positive effects (14%), and no effect (13%) (Fig. 2E). A mosaic plot of standardized residuals for the food security parameters by overall effect (Fig. 3) revealed larger counts of papers than expected with negative effects for studies examining the food security parameter of food price, positive effects for studies examining food availability, and mixed positive and negative effects for studies examining multiple food security parameters (Supplementary Fig. 9).

### Relationship of bioenergy and food security based on the sociodemographic index, spatial scale, and temporal scale

#### Sociodemographic index

**Fig. 4:**
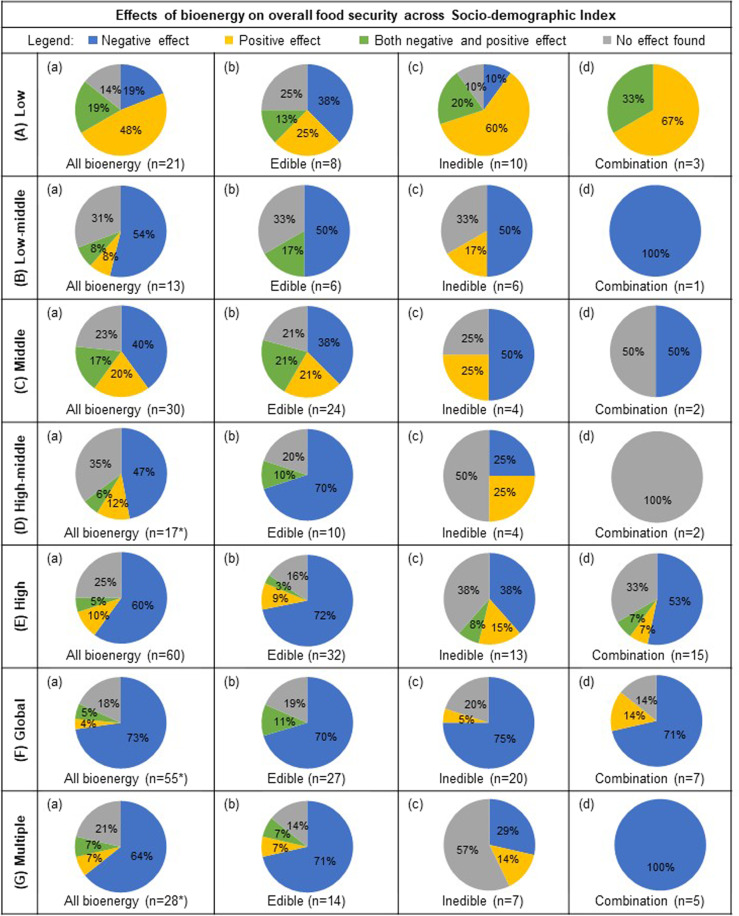
Effects of bioenergy on food security across sociodemographic index. The percentage of reviewed publications (*n* = 224) that reported either negative, positive, both negative and positive, or neutral effect of all bioenergy (column **a**), edible (**b**), inedible (**c**), and studies that included a combination of both edible and inedible feedstocks in their analysis (d) in countries with an SDI Index level (row **A**) low, (**B**) low-middle, (**C**) middle, (**D**) high-middle, (**E**) high, (**F**) global studies (all SDI levels), and (**G**) studies that included multiple SDI’s. *Bioenergy type “not specified” (*n* = 4) is only included in “All bioenergy” (column **a**), and therefore the sum of edible, inedible, and both edible and inedible feedstocks (columns **b**–**d**) will not necessarily be a composite of “All bioenergy”.

For each study location, the respective sociodemographic index (SDI) was recorded and tabulated based on the reported effects of bioenergy on overall food security parameters (Fig. 4). Several studies were global in nature and therefore accounted for all SDI levels (*n* = 55), in addition to several studies that included multiple countries and SDI levels (*n* = 28). With the exception of low SDI, bioenergy production was found to generally have a negative impact on overall food security parameters on the basis of SDI (40–73%) (Fig. 4a: B–G). Both the parametric and nonparametric chi-squared tests indicate strong evidence regarding the relationship between bioenergy production and overall food security on the basis of SDI level (*χ*^2^ = 47.5, permutation-based *P* value = 0.001). Specifically, the mosaic plot of standardized residuals for the SDI group by overall effect revealed that studies examining bioenergy in low SDI countries had a greater amount of positive effects than expected, while studies in low-middle SDI countries had fewer negative effects than expected (Supplementary Fig. 11). For all levels of SDI, negative effects of bioenergy were most common; however, for global (all SDI’s), high, or multiple SDI levels, the probability that a study reported a negative effect was highest, and the probability that a study reported a positive effect was highest for the low SDI level.

### Spatial scale

**Fig. 5:**
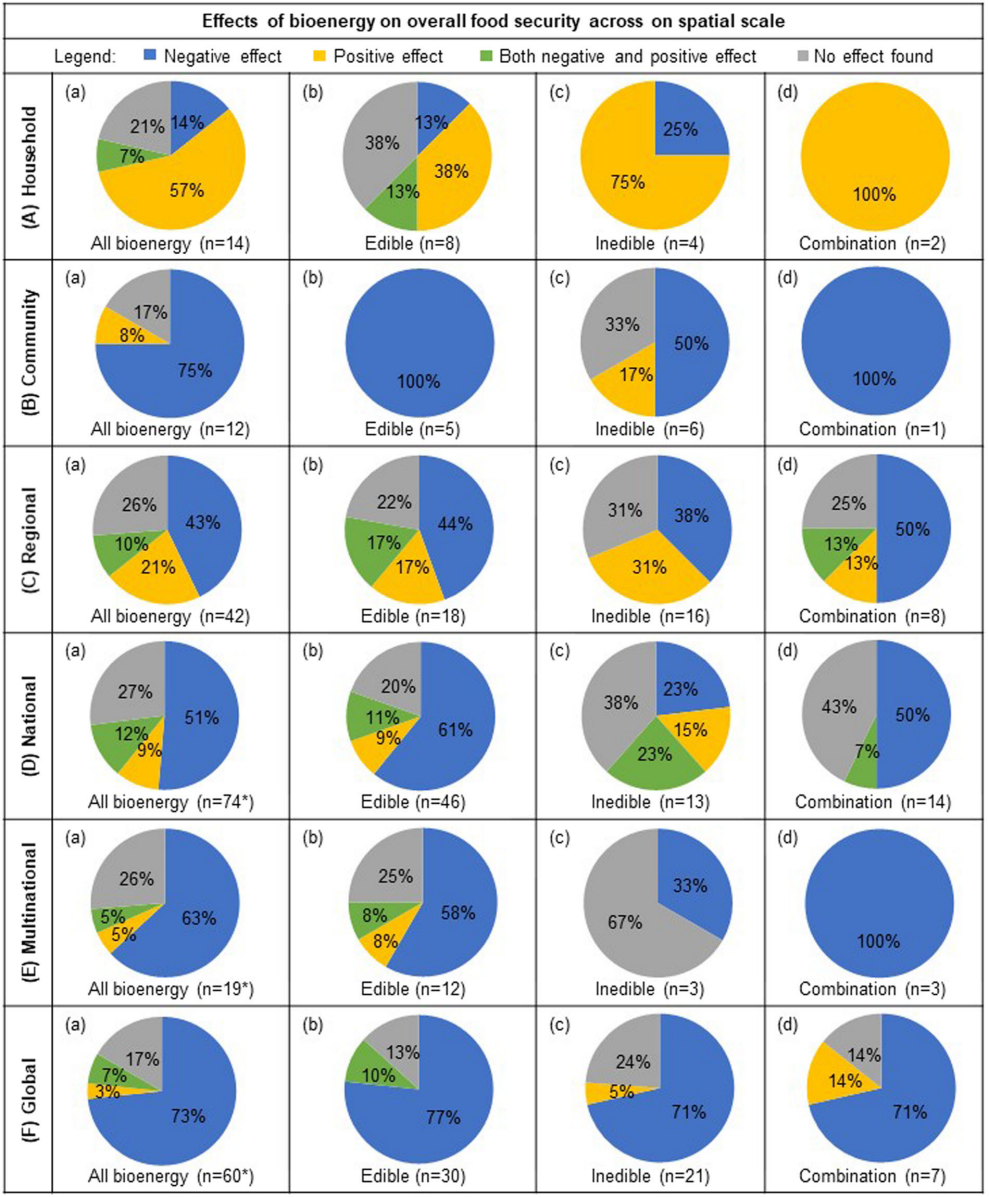
Effects of bioenergy on overall food security across the spatial scale. Percentage of peer-reviewed publications (*n* = 224) that reported either negative, positive, both negative and positive, or neutral effect of all bioenergy (column **a**), edible (**b**), inedible (**c**), and studies that included a combination of both edible and inedible feedstocks in their analysis (**d**) at the household (row **A**), community (**B**), regional (**C**), national (**D**), multinational (**E**), and global scales (**F**). *Bioenergy type “not specified” (*n* = 4) is only included in “All bioenergy” (column **a**), and therefore the sum of edible, inedible, and both edible and inedible feedstocks (columns **b**–**d**) will not necessarily be a composite of “All bioenergy”. Studies with more than one spatial scale (*n* = 3) are not included.

The reviewed studies were completed with datasets at a variety of spatial scales including household, community, regional, national, multinational, global, and a few publications that examined multiple spatial scales (Fig. 5). The majority of studies reported negative effects of all bioenergy types on overall food security parameters on the basis of spatial scale (43–75%), with the exception of the household scale, where positive effects were most prevalently reported (57%). Both the parametric and nonparametric chi-squared tests indicate strong evidence regarding the relationship between bioenergy production and overall food security on the basis of spatial scale (*χ*^2 ^= 47.7596, permutation-based *P* value <0.001). A mosaic plot of standardized residuals for the spatial scale by overall effect reveals that for studies at the household spatial scale, there were more studies that indicated a positive overall effect than would be expected and fewer negative overall effects were much smaller than expected (Supplementary Fig. 13).

### Temporal scale

**Fig. 6:**
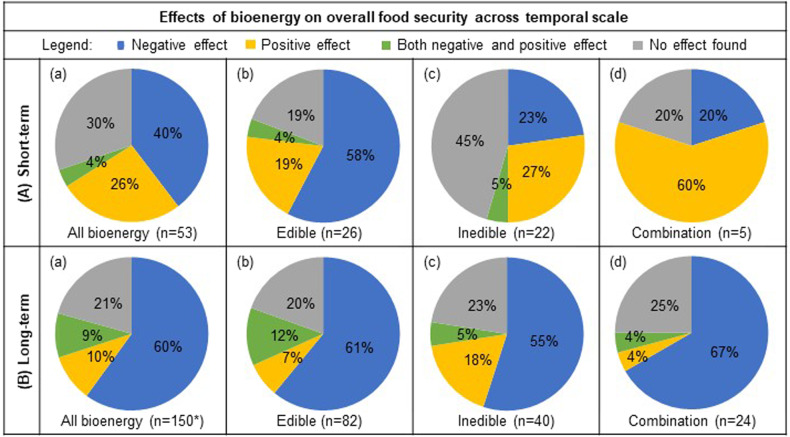
Effects of bioenergy on food security across the temporal scale. Percentage of reviewed publications that reported either negative, positive, both negative and positive, or neutral effect of all bioenergy (column **a**), edible (**b**), inedible (**c**), and studies that included a combination of both edible and inedible feedstocks in their analysis (**d**) from short-term (row **A**) and long-term scales (**B**). *Bioenergy type “not specified” (*n* = 4) is only included in “All bioenergy” (column a), and therefore the sum of edible, inedible, and both edible and inedible (columns **b**–**d**) will not necessarily be a composite of “All bioenergy”. Studies that included a combination of both short- and long-term scales, or where the temporal scale was not specified, are not included in the figure.

The reviewed publications varied on the basis of temporal scale including short-term studies with the data collected over a period of less than 6 years and long-term studies with data collected for 6 or more years (Fig. 6). Both the parametric and nonparametric chi-squared tests indicate strong evidence regarding the relationship between bioenergy production and overall food security on the basis of temporal scale (*χ*^2^ = 19.16, permutation-based *P* value = 0.017). The mosaic plot of standardized residuals for the temporal scale by overall effect revealed there were more studies that included short-term datasets and positive overall effects than would be expected if there was no relationship between temporal scale and overall effect on food security parameters (Supplementary Fig. 15). The majority of publications that examined short-term datasets reported negative effects (40% of 53 publications), followed by no effect (30%), positive effects (26%), and a few that reported a combination of both positive and negative effects (4%) (Fig. 6A). Conversely, studies that included the examination of long-term datasets more prevalently reported negative effects (60% of 150 publications) while the remaining reported no effect (21%), positive effects (10%), or both negative and positive effects (9%) (Fig. 6B). When the temporal scale is compared across the various spatial scales, publications that examined short-term datasets that reported negative results (40%) are more prevalently at the national (43%) scale, while studies that reported no effect (30%) are prevalent at the regional (38%) and national (31%) scale, and positive effects (26%) are reported at the household (43%) or regional (43%) scale. Long-term datasets with negative results (60%) are more prevalent at the global (42%) or national (28%) scale, while no effect (21%) is reported at the national (39%) and global (29%) scale and positive effects (10%) are reported at the national (40%) or regional (20%) scale. There are no positive effects reported for datasets that include a combination of both short- and long-term scales, and those with the temporal scale not specified.

### Type of the data: observed versus modeled studies

**Fig. 7:**
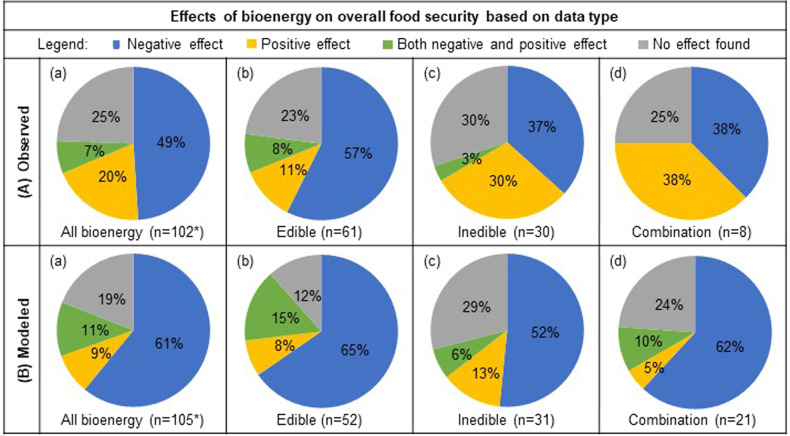
Effects of bioenergy on overall food security based on observed data and modeled data. Percentage of reviewed publications that reported either negative, positive, both negative and positive, or neutral effect of all bioenergy (column **a**), edible (**b**), inedible (**c**), and studies that included a combination of both edible and inedible feedstocks in their analysis (**d**) using observed data (row **A**) and modeled datasets (**B**). *Bioenergy type “not specified” (*n* = 4) is only included in “All bioenergy” (column a), and therefore the sum of edible, inedible, and both edible and inedible feedstocks (columns **b**–**d**) will not necessarily be a composite of “All bioenergy”. Studies that used a composite of both observed and modeled data (*n* = 17) are not included in the figure.

Analysis of studies based on the type of the data (observed and modeling studies) (Fig. 7) revealed that almost half the studies using observed data reported negative effects (49% of 102 publications), while a quarter found no effect (25%), fifth reported positive effects (20%), and a few reported both negative and positive effects (7%) (Fig. 7A). The majority of studies using modeling for predicting effects of bioenergy on food security parameters reported negative effects (61% of 105 publications) while approximately one-fifth found no effect (19%) and a few reported both a negative and positive effect (11%) as well as positive effects (9%) (Fig. 7B). For the studies utilizing both observed data and modeling, the majority reported negative effects of bioenergy on food security (65% of 17 publications) while the remaining third reported no effect (35%). Statistical analysis revealed moderate evidence regarding the relationship of bioenergy and food security on the basis of the type of data (*χ*^2^ = 13.47, permutation-based *P* value = 0.036). The mosaic plot of standardized residuals for the dataset type by overall effect revealed studies using observed datasets reported fewer negative effects on food security and the studies using modeled data reported more negative effects on food security than expected (Supplementary Fig. 17).

### Statistical learning: relationship of bioenergy and food security

**Fig. 8:**
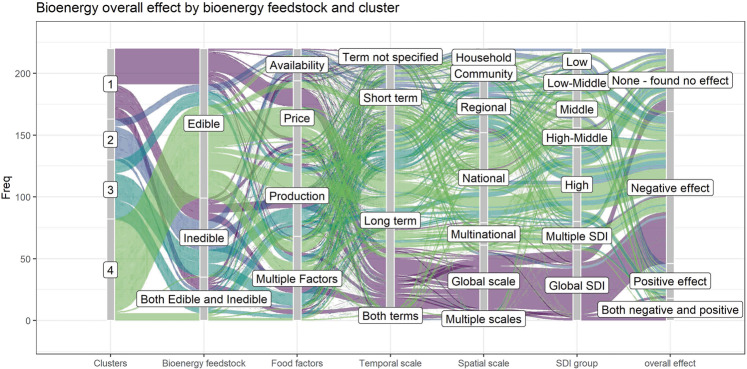
Cluster characteristics and cluster descriptions. Alluvial plot flowing left to right of clusters one through four identified followed by the type of bioenergy, the food factors and the overall effect of the bioenergy on food security from a review of 220 journal articles. *Bioenergy type “not specified” was not included in the cluster analysis.

Hierarchical cluster analysis with Ward’s method on Gower’s dissimilarity identified four clusters on the basis of the type of bioenergy feedstock (edible versus inedible feedstocks), food security parameters, temporal and spatial scales, and SDI levels with the following characteristics: (i) cluster one had the highest proportion of negative effects of bioenergy; (ii) cluster two had the highest proportion of positive or no effect of bioenergy; (iii) cluster three had the highest proportion of both negative and positive effects of bioenergy; (iv) cluster four had a high proportion of negative effects or no effect of bioenergy (Fig. 8); additional details and visualizations of results are provided in Supplementary Methods (Supplementary Figs. 19–21).

Three random forest models fit based on food security parameters (food availability, food price, and food production) (Supplementary Methods and Supplementary Figs. 22–24)) in the machine-learning approach revealed that papers that examined the food security parameter of food availability at the household scale were more likely to report positive effects of bioenergy on overall food security. In addition, papers that examined low-middle SDI countries at the community scale were more likely to report a negative effect of bioenergy on overall food security. With regards to the food security parameter of food price, papers that examined edible feedstocks, or at a global scale were more likely to report a negative effect of bioenergy production on overall food security. Further, papers that examined food price and included middle SDI countries were more likely to report both no effect and both negative and positive effects. Finally, papers that examined food prices that included low SDI countries, or that examined the household or regional scale were associated with finding no effect of bioenergy on food security. Regarding the food security parameter of food production, papers that included low SDI countries or examined food security at the household scale were more likely to report positive effects of bioenergy on overall food security.

## Discussion

### Relationship of bioenergy and food security

Achieving food security is a critical challenge of the Anthropocene that may conflict with the provision of ecosystem services and other societal goals related to the UN Sustainable Development Goals (SDGs) for increased energy access and combatting climate change. The potentially detrimental effects of bioenergy expansion on food security as captured in the “fuel versus food” debate coupled with efforts to mitigate climate change have given rise to next-generation biofuels including those produced from feedstocks that are inedible. This systematic review contributes to the “fuel versus food” debate by synthesizing the totality of the evidence regarding the effects of bioenergy feedstocks that are classified on the basis of edibility for human and livestock consumption on various food security parameters and, how this relationship varies on the basis of the sociodemographic index, spatial and temporal scale, and data type.

Over half the publications identified in this systematic review (56% of 224 publications) reported a negative effect of bioenergy on food security parameters (food availability, food price, food production, and multiple food security parameters). The overall effects of bioenergy on food security were not significantly different on the basis of the edibility of the feedstocks examined for human and livestock consumption (*P* value = 0.15). However, a strong relationship was found between bioenergy and type of food-security parameter (*P* value < 0.001) as well as on the basis of spatial scale on which food security was measured (*P* value < 0.001) and the sociodemographic index (SDI) of the study location (*P* value = 0.001).

### Food-security parameters, scale, and SDI

The findings of this systematic review emphasize the importance of examining multiple parameters of food security at various spatial and temporal scales. While bioenergy expansion may have positive effects on food security based on one parameter or scale, the response of food security may be different based on another parameter or a different scale. For example, food prices were found to be the most vulnerable parameter in response to bioenergy production with regards to negative outcomes.

On the basis of scale, food security at the household scale was found to be less vulnerable compared to the community, national, or global levels while a greater number of studies reported negative implications of food security for the long term. The positive effect of cultivating bioenergy feedstocks at the household scale demonstrates the local importance that bioenergy feedstocks can play in diversifying the income of small-holder farmers and in providing them with a crop that provides a relatively high price. In contrast to this positive local effect, the negative effects of bioenergy feedstock production at larger scales may be due to higher prices faced by consumers and competition of feedstocks with other food and feed crops.

In addition, the evidence synthesized in this review suggests that the effects of bioenergy production on food security may vary over time with accumulating negative effects; ~40% of the short-term studies reported negative effects compared to 60% of the long-term studies. Findings further highlight the variable implications of bioenergy production for food security on the basis of sociodemographic factors characterizing a particular location. The response of bioenergy is not the same across countries with those classified with a high SDI most likely to experience negative implications for food security. In addition, there is a need to further investigate the modeled datasets used in predicting the effects of bioenergy to ground-truth these outcomes.

### Beyond food security: multiple sustainable development goals toward planetary health

This systematic review provides evidence of the importance of the integration of bioenergy and food production for supporting food security. For example, Egeskog et al. reported that agricultural systems in Brazil that integrated both dairy and ethanol production could benefit from sugarcane residues used to feed cattle as well as a tenfold increase in income^47^. In an organic system, Paulsen found that bioenergy expansion in oilseeds with legumes or cereals has the potential to provide on-farm fuel needs while simultaneously producing food crops^48^. In addition to sociodemographic factors, several studies in this review also point to the importance of infrastructure, technology, and policy in determining the implications of bioenergy on food security in a given context. For example, in locations with high biomass production and technology and infrastructure in place, millions of tonnes of biomass from agricultural residues can be used for biofuel without affecting food production, such as certain areas in Africa reported by Chimphango et al.^49^, though the conversion of biomass to biofuel may be hindered by economic feasibility^26^ Habib-Mintz reported that in the absence of policy or regulatory frameworks for biofuel development, biofuels produced at an industrial scale from *Jatropha* in Tanzania could exacerbate both poverty and food insecurity^50^.

While the overall effects of bioenergy on food security outcomes were not significantly different on the basis of the edibility of the feedstocks examined, it is important to recognize the multiple implications of bioenergy production for achieving other environmental and societal goals of the SDGs that were not examined in this systematic review. The feedstocks that were classified as edible for human and livestock consumption on the basis of our edibility schematic are all classified as first-generation biofuels according to the IPCC classification scheme and none are classified as second-generation and third-generation biofuels^44^. In contrast, feedstocks that were classified as inedible included some first-generation as well as second- and third-generation biofuels. Despite the absence of a difference between edible and inedible feedstocks in reducing conflicts between bioenergy production and food security, inedible first-generation biofuels (such as waste oils or *Jatropha*), as well as next-generation biofuels produced on marginal or degraded land, may have other positive environmental and societal benefits such as biodiversity conservation and continued provision of ecosystem services and mitigating climate change^21^. However, the conversion process to biofuel may be associated with higher costs and variable economic feasibility dependent on feedstock^26^. Indirectly, these benefits such as biodiversity conservation and the continued provision of ecosystem services have the potential to ameliorate conflict with food security^51^. In addition to bioenergy and food security interactions synthesized here, the implementation of bioenergy strategies for climate mitigation can negatively or positively impact other parameters of planetary health such as biodiversity and water resources^52,53^, and loss of ecosystem services through land-use change^54^ and thus need to also be examined. This points to the need for programs and policy to adopt an integrative and multifaceted framework such as the food–water–energy–biodiversity–social systems (FWEBS) framework to monitor trade-offs and identify cross-sector efficiencies in meeting societal demands and addressing challenges^21^.

## Conclusion

Overall, findings suggest a critical need for programs and policies focused on bioenergy expansion to take an integrative and long-term approach to coordinate multiple sustainability goals. Increased energy demands are directly interwoven with food systems with both risks and opportunities in economic, social, and environmental dimensions, depending on context^55^. In order to be a feasible alternative to fossil fuels, bioenergy production should provide a balance of energy, environmental, and economic benefits, while not competing for food security either directly or indirectly. Within both edible and inedible feedstocks, numerous trade-offs exist among environmental and economic costs associated with cultivation, energy efficiency and greenhouse gas emissions, and production costs^26^. However, evidence synthesized in this review highlights that the reported effects between edible and inedible feedstocks on food security are not significantly different.

Our specific recommendations for the alignment of bioenergy and food security are as follows:

1. Bioenergy projects should monitor food security on the basis of multiple food security parameters as well as spatial scales over the long-term.
2. Given the issues of comparing food security outcomes across studies, there is a need to design more widely agreed upon international standards of multiple dimensions of food security in addition to household-level food security that is culturally relevant in different contexts. The development of such measures should be based on long-term global collaborative projects with congruent experimental designs and “big data” sharing to allow for comparison of food security outcomes over time.
3. There is a need to foster multisectoral engagement between resource managers, communities, enterprises, scholars, and policymakers to examine the trade-offs of bioenergy expansion for multiple societal goals for supporting multiple Sustainable Development Goals in addition to food security including biodiversity, ecosystem services, climate mitigation, and cultural implications^21,26^. Such multisectoral engagement should adopt integrative frameworks to monitor trade-offs and identify cross-sector efficiencies in meeting societal demands and addressing challenges^21^. In addition, these projects should evaluate the arability of land used to produce biofuels since first-generation and next-generation biofuels can be produced on both arable and marginal/degraded lands.
4. Policy and regulatory frameworks are needed in order to govern the implications of bioenergy expansion for multiple societal goals including supporting incentives for the integration of bioenergy feedstock production and food crops as part of sustainably managed mixed-crop systems.
5. Investments should support technology and infrastructure that can ameliorate the detrimental impacts of bioenergy production on food security.

## Methods

### Setting up the systematic literature review

The design of this study applies the Preferred Reporting Items for Systematic Reviews and Meta-Analyses (PRISMA)^56^ and the Guidelines for Systematic Review and Environmental Synthesis in Environmental Management^57^ to collect evidence on the closed-frame study question: what are the effects of various classes of bioenergy feedstocks on food security parameters (food availability, food prices, and food production)? Given our emphasis on food security, we classified bioenergy feedstocks as being edible versus inedible and specifically addressed: what are the effects of bioenergy feedstocks classified as edible versus inedible on food security parameters (food availability, food prices, and food production)? We examine how this relationship varies on the basis of the sociodemographic index, spatial and temporal scale, and data type. The Population, Intervention/Exposure, Comparator, Outcome (PICO)^58^ framework elements were used to structure this systematic review with bioenergy production as the intervention/exposure and food security as the outcome.

Search terms for the review protocol were identified by a panel of experts in the fields of food security, bioenergy, and global change. A selected group of search terms were tested in databases to find relevant literature and were revised through an iterative process to address the study question as well as feasibility for conducting a manageable systematic review (Supplementary Table 20). The final set of search terms was entered into six publication databases: Web of Science, EBSCO GreenFILE, Agricola, PubMed, ProQuest, and Science Direct. Inclusion criteria included all primary, peer-reviewed publications published in the English language from January 1, 2000 to September 10, 2017. Primary (or original) research publications included in this study were defined as those that reported results using either primary data (observed field data) or available data sources to conduct original data analysis (modeling studies). Concept papers, conference proceedings, reviews, and other published work that did not involve original data analysis were excluded.

### Literature screen and full-text review

**Fig. 9:**
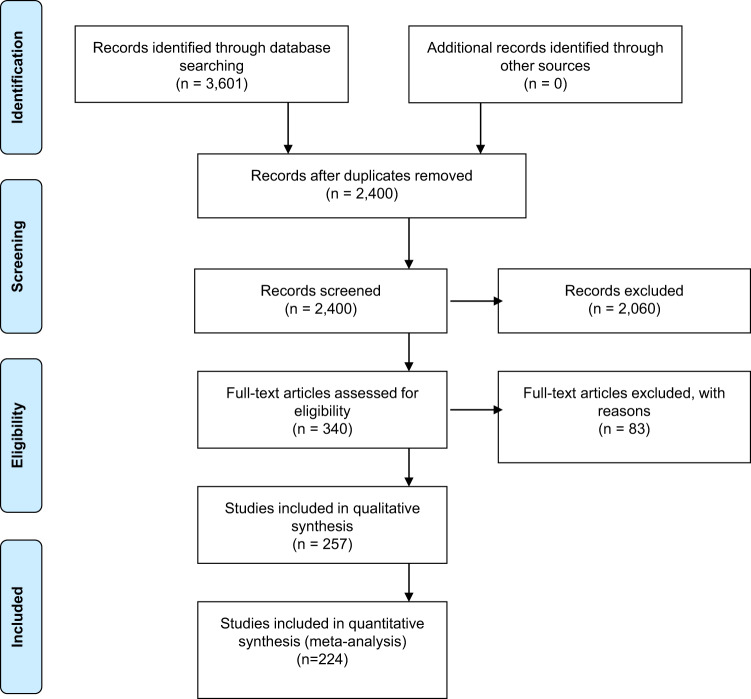
PRISMA flow diagram outlining the systematic review process. The Preferred Reporting Items for Systematic Reviews and Meta-Analyses (PRISMA) Flow Diagram^56^ outlines the screening process from the number of publications identified through the database search to the final number of publications included in the qualitative synthesis that met the a priori inclusion criteria.

The Covidence software program (of the Cochrane Technology Platform) was used to manage and screen publications matching the a priori inclusion criteria by a screening panel of seven reviewers. The title and abstract of each publication were screened by two reviewers from the review panel to minimize reviewer bias in identifying publications for their relevance. Discrepancies in inclusion resulting from the screening process were discussed by at least two reviewers on the review panel to resolve conflicts between reviewers. Publications with abstracts that met the inclusion criteria proceeded to the full-text screening process, with all other publications excluded from the review. The review panel critically appraised publications identified to meet the search criteria during the full-text screen to validate their inclusion (Fig. 9).

### Qualitative data analysis

The following information was extracted from the final set of publications from the full-text screen by a member of the screening panel (definitions proceeding): (1) publication title, (2) publication author(s) and year, (3) location and sociodemographic index, (4) bioenergy feedstock examined, (5) food security parameter(s) measured, (6) spatial scale, (7) temporal scale, (8) observed or modeled data, and (9) summary of effects of bioenergy production on food security parameters (Supplementary Table 1). Study location included country or global scale for each study, and the respective sociodemographic index level for each country. Sociodemographic index (SDI) is a measure of the development of a country correlated with health outcomes, quantified by an index of 0 (lowest health outcome) to 1 (highest health outcome), and corresponding with a scale of SDI quintile levels from low, low-middle, middle, high-middle, and high^59^. When a study was conducted at a global scale, all SDI levels were accounted for (global SDI/all SDI levels). On the basis of the classification scheme of feedstock edibility presented in the Background, bioenergy feedstocks examined in the studies were classified as either edible, inedible, both edible, and inedible (where multiple feedstock types were examined), or not specified. Food-security parameters were categorized as either food availability, food prices, food production, or multiple food security parameters (for studies that included a combination of food availability, food price, and food production). Given the multidimensionality of food security parameters, we were not able to compare studies on the basis of units. For example, food availability included aspects affecting the overall availability of food, such as food insecurity, current, and potential food access/availability, farmer livelihoods, and risk of hunger. Food price included aspects affecting the price of food such as the rise and/or decrease in food prices for consumers and producers, absolute price, price volatility, price elasticity, animal feed prices, food and land prices, commodity markets, and cost of diets. Finally, food production included aspects affecting the production of food such as food crop displacement due to land-use change, food production processes, manufacturing, exports in multiple agricultural sectors, effects of inter-cropping on food production, stakeholder perceptions regarding the effect of bioenergy expansion on food production, and reductions in resources needed for food production, including land, water, and fertilizer. The spatial scale of the study within each publication examined was classified at the following levels: household, community, regional (region within a country), national, multinational, global, and those with “multiple” scales examined. Household-level scales considered the effect of bioenergy production for an individual household, while the effects at the national, multinational, and global level include effects across an increasingly vast spatial scale. The temporal classification of studies within each publication included either short-term studies with data collected over a period of <6 years, or long-term studies with data collected 6 or more years. Studies were further classified as using observed field data, or those using predicted and/or modeled data analyses of bioenergy expansion with secondary data sources. Finally, a qualitative synthesis of the effects of bioenergy production on food security parameters was summarized for each publication.

A panel of three reviewers per study read the outcome summary for each publication and classified the study as having the following outcomes or effects of bioenergy production on food security: (1) negative effect; (2) positive effect; (3) both negative and positive effects; and (4) no effect (neutral effect). Negative effects of bioenergy on food security were classified based on evidence that indicated any reduction in the availability of food experienced by a small-holder, increased food prices for consumers, increased volatility of food prices, and any reduction in food production which would translate to reduced producer income. Conversely, positive effects of bioenergy on food security were classified based on evidence from the studies that indicated increased food availability, reduced food prices for consumers, and increased food production or increased producer income. In cases where both negative and positive effects of bioenergy on food security were found, the effects were accounted for as “both negative and positive effect”. For example, reduced food price for consumers was classified as a positive effect while the reduced price for producers was classified as a negative effect. Alternately, increased food price for consumers was classified as a negative effect while increased prices for producers classified as a positive effect. Finally, classification of no effect was generally used in scenarios such that if certain conditions were met, there would not be an impact on food security. For example, in a future projection scenario, if inedible feedstocks were produced on marginal land, there would be no impact in food security^60^. In another example, the production of *Jatropha* may reduce small-holder food production, however food availability is increased due to an increase in small-holder income^61^. The panel discussed discrepancies in their classification regarding the effects of bioenergy production on food security parameters and came to a final decision for each study. The entirety of this information is compiled in Supplementary Table 1, which includes a rich descriptive qualitative synthesis for each publication reviewed.

### Quantitative analysis

The publications were quantitatively synthesized through tabulation of frequencies of effects of bioenergy production on *o*verall food security across attributes of food security parameters, SDI, spatial scale, temporal scale, and type of the data used in each study. The figures throughout the “Results” section present a quantitative synthesis of the effects of bioenergy production on all parameters examined across the different bioenergy feedstocks. In addition, relationships among variables with the type of bioenergy effect were assessed with parametric or permutation-based (1000 permutations used) chi-square tests, and associated mosaic plots. Two different multidimensional explorations were conducted to explore the complex relationships among the various attributes of the studies included in this systematic review (Supplementary Methods). The statistical learning methods considered three techniques to explore paper-level connections to reveal relationships among the publications. Hierarchical cluster analysis with Ward’s method on Gower’s dissimilarity based on the type of bioenergy feedstock (edible versus inedible feedstocks), food security parameters, temporal and spatial scales, and SDI levels, was used in order to identify groups of reviewed studies with similarities regarding these five features^62^. In addition, the relationships between the overall effect and the paper-level variables were extracted using a machine-learning approach. Specifically, random forest models, decision-tree ensembles in which each tree is constructed through bootstrap aggregation and sampling from the explanatory variable space^63^, were used due to their suitability for capturing complex interactions between explanatory variables. The impact of each binary indicator created from the variables (SDI, type of feedstock, spatial scale, temporal scale, and data type) was assessed through partial dependence plots^64^. All analyses were completed with R 4.0.2 using RMarkdown with original source code and dataset available upon request from the lead author; more details on statistical methods and specific packages used are in the Supplementary Methods.

## Supplementary information

Supplementary Table 1

Supplementary Material

## Data Availability

All data generated or analyzed during this study are included in this published article and Supplementary Methods.

## Code availability

The code generated to analyze the datasets during this study is available from the corresponding author on reasonable request.
