# Supplementary material for: Systematic review on effects of bioenergy from edible versus inedible feedstocks on food security
Source: NPJ Sci Food. 2021 May 4;5:9. doi: 10.1038/s41538-021-00091-6 (PMC8096942; doi:10.1038/s41538-021-00091-6)
Supplement: Supplementary file 2 — Supplementary Material [file 41538_2021_91_MOESM2_ESM.pdf]

Supplementary Table 1. All publications (n=224) included in meta-analysis of this systematic review.

Supplementary Methods

Data for the statistical analysis was provided in an Excel spreadsheet. To make it usable for R some transformations were necessary including renaming the columns to shorten names and remove spaces and to assert that categorical factor variables were identified as such.

The Bioenergy Feedstock variable (bioenergyFeedstock) levels were set to ensure that “Not Specified” was set as the baseline level and the other levels in order are “Edible”, “Inedible”, and “Both Edible and Inedible”.

The overall effect variable (overallEffect) levels were set to ensure that “None - found no effect” was set as the baseline level and the other levels in order are “Negative effect”, “Positive effect”, and “Both negative and positive”.

The binary variables for food security parameters (foodPrice foodProd, and foodAvail) were combined into one categorical factor variable foodFactors with possible values of “Availability”, “Price”, “Production” and “Multiple Factors”.

The binary variables for SDI groups (globalSDI, lowSDI, lowMiddleSDI, midSDI, highMidSDI, highSDI) were combined into one categorical factor variable SDIgroup with possible values of “Low”, “Low-Middle”, “Middle”, “High-Middle”, “High”, “Multiple SDI” and “Global SDI”.

All work was completed using RMarkdown from the original data set and the file and dataset to reproduce all analyses are available upon request.

An Alluvial diagram (Brunson, 2020) was used to visualize the relationships between the variables (1) Bioenergy Feedstock designation, (2) SDI group, (3) Food factors (also referred to as food security parameters), and (4) Overall effect. Here, the Alluvial diagram indicates the proportion of the total for all of the categories for each variable and traces how many observations move from one factor level to the next. The colors represent the overall effect of bioenergy with “None - found no effect”, “Negative

effect", "Positive effect" and "Both negative and positive" represented by purple, blue, green and yellow-green respectively.

The first alluvial diagram (Supplementary Figure 1) includes all 224 journal articles. We can trace the flows backwards (right to left) to see how the bioenergy overall effect is related to the Bioenergy feedstock, SDI group, and food factors. We can see that the majority of the articles that indicated a negative overall effect are in the food price, food production and multiple food factor levels, and are spread across all SDI groups, although more heavily concentrated in the higher SDI, multiple or global SDI groups and roughly equal in all bioenergy feedstocks. The articles that found no effect indicated food production and there were various SDI groups as well as bioenergy feedstocks. Positive overall effects were not found in papers that indicated food price was a factor and were more often in lower SDI groups. Both negative and positive overall effects were more likely found in papers with multiple food factors and varying SDI groups and bioenergy feedstocks.

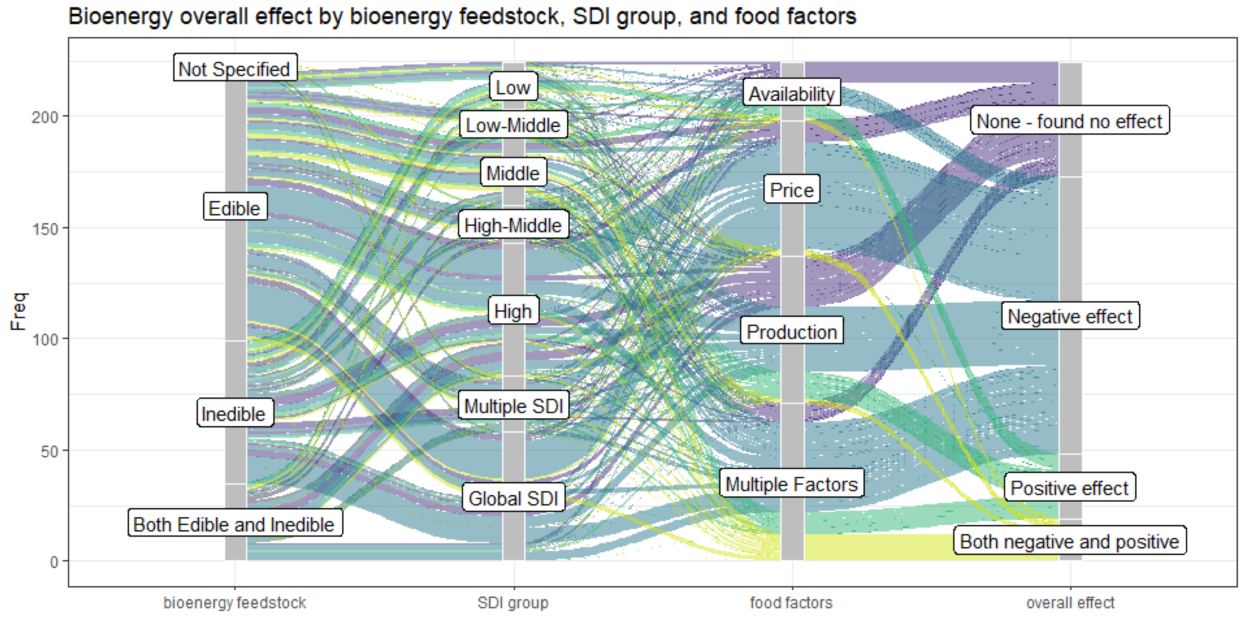

Supplementary Figure 1: Alluvial plot of the bioenergy feedstock, the SDI group, the food factors and the overall effect of the bioenergy on food security from a review of 224 journal articles.

As there were only four papers with bioenergy feedstock not specified, these papers were removed from the dataset and a new alluvial diagram generated. The patterns visible did not drastically change with the removal. However, the papers that discussed edible bioenergy with a low SDI group were more easily visible with the removal of those four papers.

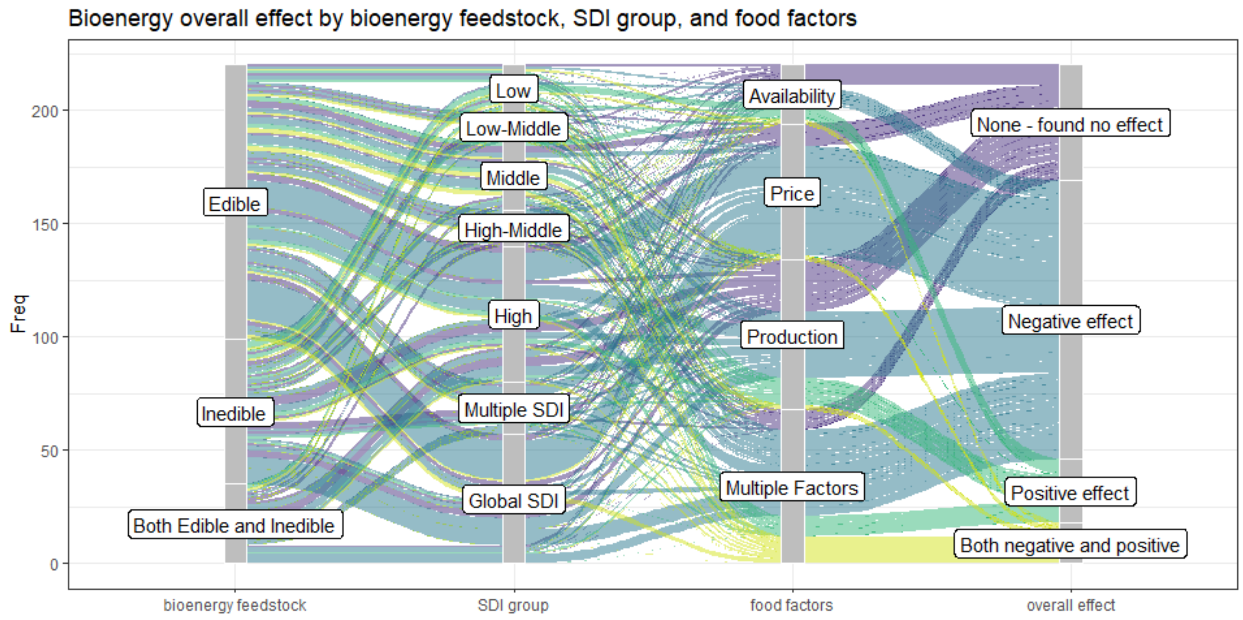

Supplementary Figure 2: Alluvial plot of the bioenergy feedstock, the SDI group, the food factors and the overall effect of the bioenergy on food security from a review of 220 journal articles after removing the four articles with bioenergy feedstock not specified.

The final alluvial diagram simplifies the variables used down to food factors, bioenergy feedstock (Edible and Inedible separately) as well as the bioenergy overall effect. With this diagram we can again trace the flow backwards (right to left) to visualize the proportions of papers that mentioned the different food factors as well as the bioenergy feedstocks based on the bioenergy overall effect.

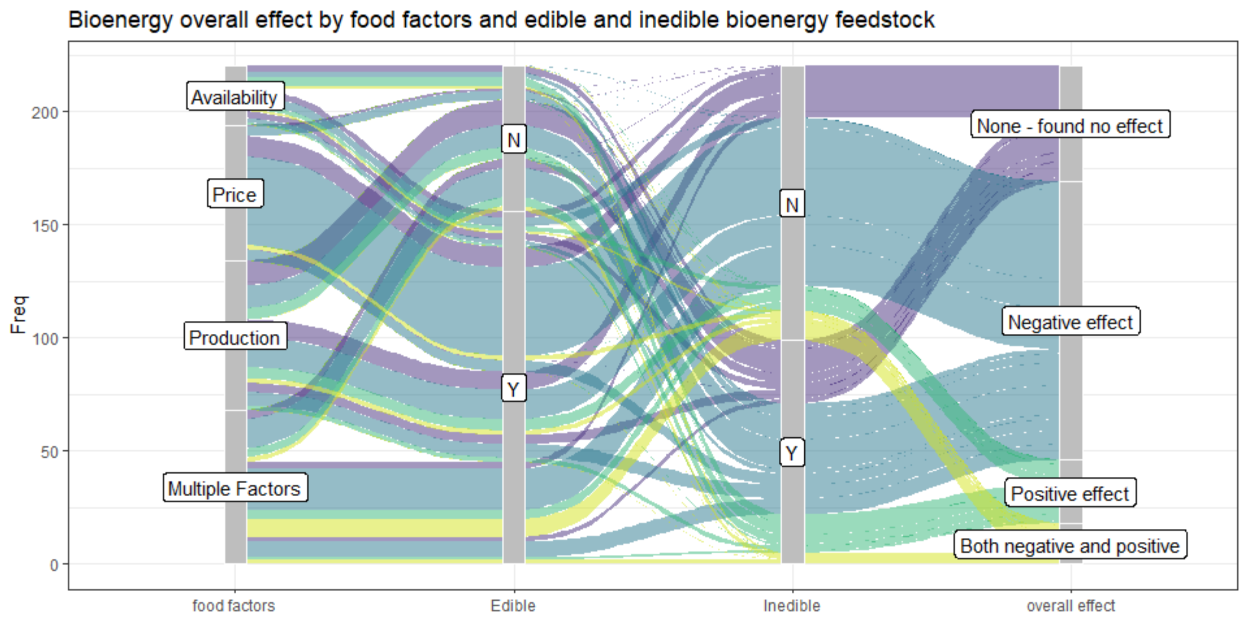

Supplementary Figure 3: Alluvial plot of the food factors, the bioenergy feedstock edible and inedible binary variables, and the overall effect of the bioenergy feedstock on food security from a review of 220 journal articles after removing the four articles with bioenergy feedstock not specified.

To summarize the data, tables were created that included counts of papers by food factor, bioenergy feedstock by overall effect on food security.

Supplementary Table 2: Counts of journal articles for each bioenergy feedstock classification by bioenergy overall effect when food availability as a food factor was mentioned in the article.

	Edible	Inedible	Both Edible and Inedible
None - found no effect	3	3	3
Negative effect	4	2	2
Positive effect	2	4	1
Both negative and positive	1	1	0

Supplementary Table 3: Counts of journal articles for each bioenergy feedstock classification by bioenergy overall effect when food price as a food factor was mentioned in the article.

	Edible	Inedible	Both Edible and Inedible
None - found no effect	9	1	0
Negative effect	39	4	5
Positive effect	0	0	0
Both negative and positive	2	0	0

Supplementary Table 4: Counts of journal articles for each bioenergy feedstock classification by bioenergy overall effect when food production as a food factor was mentioned in the article.

	Edible	Inedible	Both Edible and Inedible
None - found no effect	8	11	4
Negative effect	13	10	6
Positive effect	5	5	2
Both negative and positive	2	0	0

Supplementary Table 5: Counts of journal articles for each bioenergy feedstock classification by bioenergy overall effect when multiple food factors were mentioned in the article.

	Edible	Inedible	Both Edible and Inedible
None - found no effect	0	0	0
Negative effect	0	0	0
Positive effect	0	0	0
Both negative and positive	0	0	0

To complete the statistical analysis, we first start with pairwise Chi-Squared analysis to see if there is a relationship between a few of the factor variables on the overall effect of food security as reported by the 224 journal articles. For example, to understand the overall effect by bioenergy feedstock, all feedstock designations (Edible, Inedible, Both Edible and Inedible, and Not Specified) were included in the model.

Supplementary Table 6: The number of journal articles by bioenergy feedstock and overall effect on food security.

	Not Specified	Edible	Inedible	Both Edible and Inedible	Total
None - found no effect	0	23	19	9	51
Negative effect	2	74	29	20	125
Positive effect	1	11	13	4	29
Both negative and positive	1	13	3	2	19
Total	4	121	64	35	224

Pearson's Chi-squared test

```
data:  bioenergyGenByEffect
X-squared = 13.062, df = 9, p-value = 0.1598
```

Supplementary Table 7: Chi-Squared expected cell counts if there is no relationship between bioenergy feedstock and overall effect on food security.

	Not Specified	Edible	Inedible	Both Edible and Inedible
None - found no effect	0.9107143	27.54911	14.571429	7.96875
Negative effect	2.2321429	67.52232	35.714286	19.53125
Positive effect	0.5178571	15.66518	8.285714	4.53125
Both negative and positive	0.3392857	10.26339	5.428571	2.96875

Since there are several categories with small counts (less than 5) and expected cell counts were lower than required for the parametric test, primarily in the bioenergy feedstock “Not Specified” category and the “Both negative and positive” overall effect category here, a non-parametric (permutation-based) Chi-Squared test was used to test for this relationship. More details on the permutation test used here can be found in (Greenwood (2020)). A randomization seed was fixed to ensure reproducibility of results.

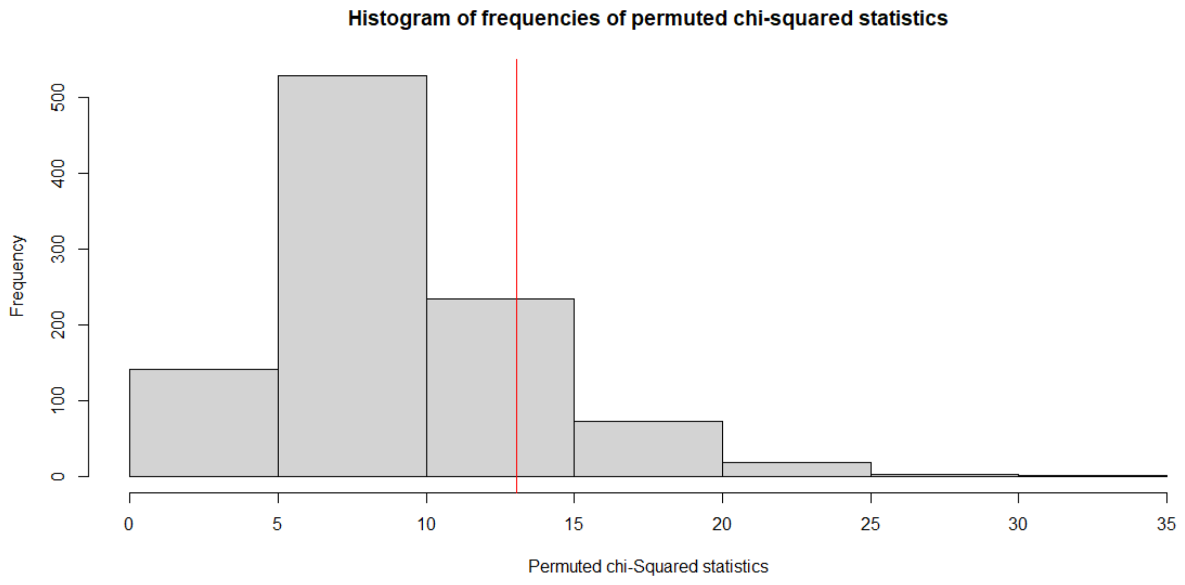

Supplementary Figure 4: Histogram of permuted test statistics for the Non-Parametric Chi-Squared test of a relationship between bioenergy feedstock and overall effect on food security. The vertical red line marks the observed Chi-Squared test statistic.

Both the parametric and non-parametric Chi-Squared test indicate there is little to no evidence against the null hypothesis of no relationship between bioenergy feedstock and the overall effect on food security ($\chi^2 = 13.0621$, permutation-based p-value = 0.152). Thus we cannot conclude that there is a bioenergy feedstock effect, or the effect could be masked because of the categories with low counts.

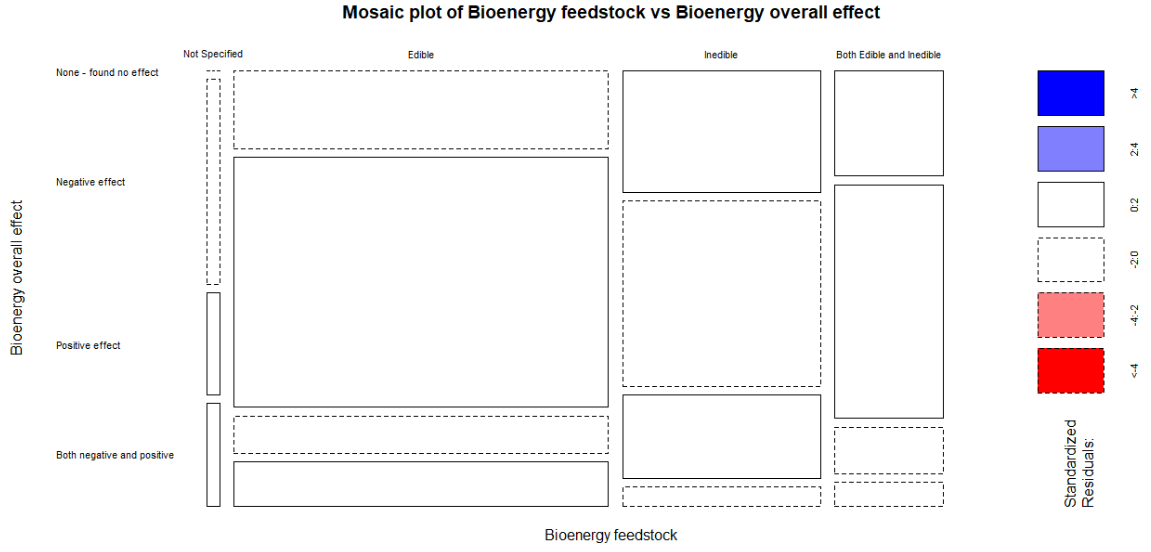

Supplementary Figure 5: Mosaic plot of the standardized residuals for bioenergy feedstock by overall effect. None of the residuals are shaded indicating that all standardized residuals are less than 2. Negative residuals are indicated with a dashed border and positive residuals are indicated with a solid border.

The mosaic plot above shows that none of the standardized residuals are greater than 2 in absolute value, indicating that none of the combinations of bioenergy feedstock and overall effect are much greater than or less than expected if there was no relationship between the bioenergy feedstock and the overall effect on food security. This is as to be expected as the statistical test also indicated that we are not able to conclude that there is a feedstock effect. This plot also helps to visualize the combinations with small cell counts compared to the more common combinations of “Negative effect” and “Edible”.

Alternately, studies were removed from the model with bioenergy feedstock “Not Specified”.

Supplementary Table 8: The number of journal articles by bioenergy feedstock and overall effect on food security after removing papers with bioenergy feedstock not specified.

	Edible	Inedible	Both Edible and Inedible	Total
None - found no effect	23	19	9	51
Negative effect	74	29	20	123
Positive effect	11	13	4	28
Both negative and positive	13	3	2	18
Total	121	64	35	220

```
  Pearson's Chi-squared test

data:  bioenergyFeedstockByEffect2
X-squared = 10.448, df = 6, p-value = 0.107
```

Supplementary Table 9: Chi-Squared expected cell counts if there is no relationship between the bioenergy feedstock and overall effect on food security.

	Edible	Inedible	Both Edible and Inedible
None - found no effect	28.05	14.836364	8.113636
Negative effect	67.65	35.781818	19.568182
Positive effect	15.40	8.145454	4.454546
Both negative and positive	9.90	5.236364	2.863636

Since there are two categories with small expected counts, in the bioenergy feedstock "Both Edible and Inedible" category, a non-parametric (permutation-based) Chi-Squared test was used to test for this relationship.

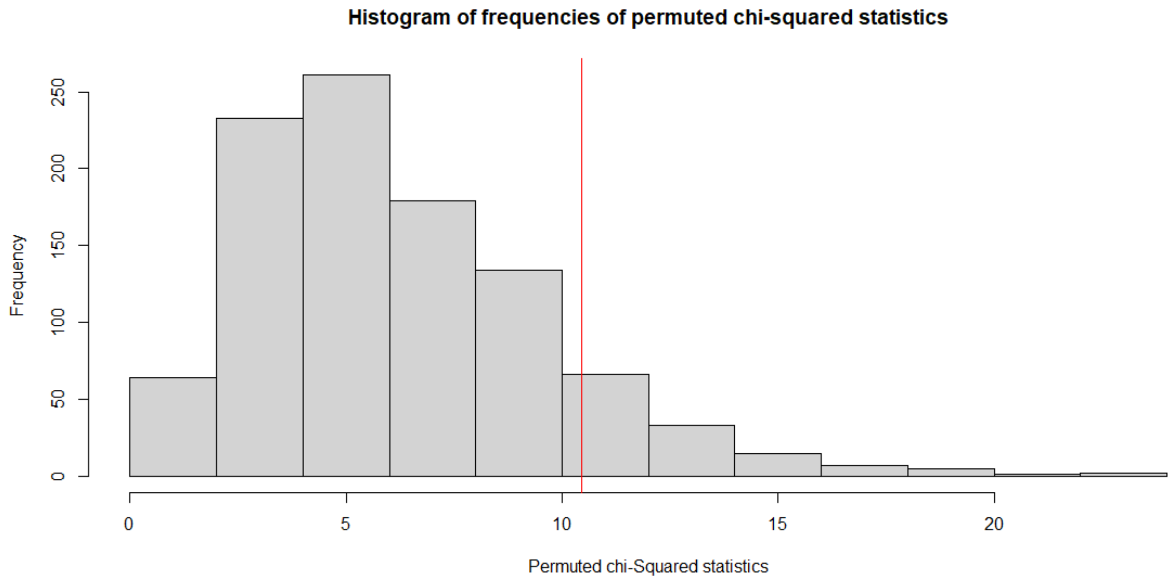

Supplementary Figure 6: Histogram of permuted test statistics for the Non-Parametric Chi-Squared test of a relationship between the bioenergy feedstock and overall effect on food security. The vertical red line marks the observed Chi-Squared test statistic.

Both the parametric and non-parametric Chi-Squared test indicate there is little to no evidence against the null hypothesis of no relationship between the bioenergy feedstock and the overall effect on food security ($\chi^2 = 10.4485$, permutation-based p-value = 0.106). Thus we can't conclude that there is a type effect, or the effect could be masked because of the categories with low counts.

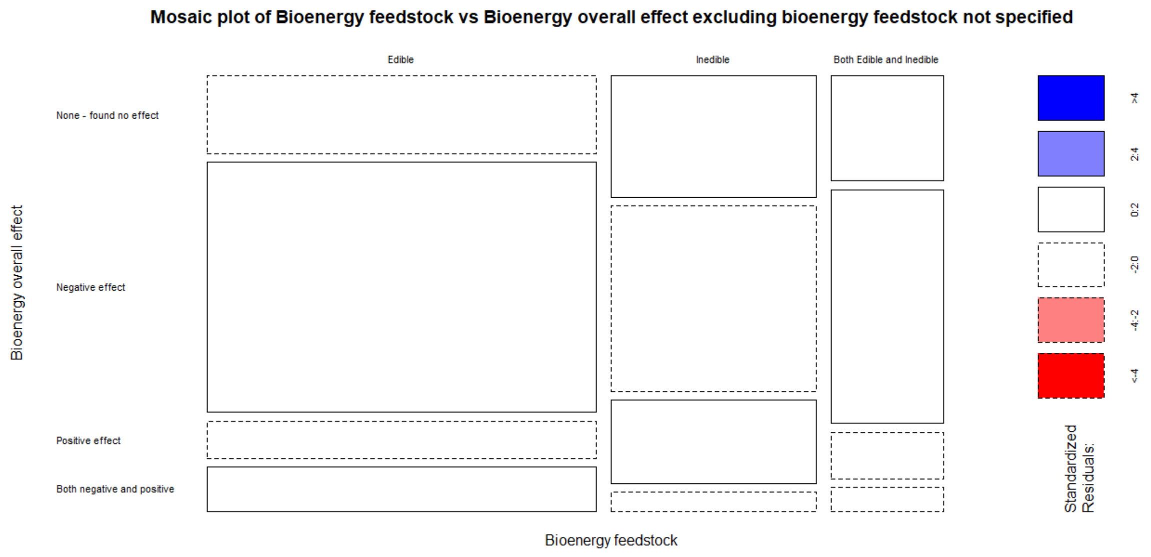

Supplementary Figure 7: Mosaic plot of the standardized residuals for the type by overall effect. None of the residuals are shaded indicating that all standardized residuals are less than 2. Negative residuals are indicated with a dashed border and positive residuals are indicated with a solid border.

The mosaic plot above shows that none of the standardized residuals are greater than 2 in absolute value, indicating that none of the combinations of bioenergy feedstock and overall effect are much greater than or less than expected if there was no relationship between the bioenergy feedstock and the overall effect on food security. This is as to be expected as the statistical test also indicated that we can't conclude that there is a bioenergy feedstock effect. This plot also helps to visualize the combinations with small cell counts compared to the more common combinations of "Negative effect" and "Edible".

Next, we analyzed overall effect by food factors (Food Price, Food Production, and Food Availability, or Multiple Factors).

Supplementary Table 10: The number of journal articles by food factors and overall effect on food security.

	Availability	Price	Production	Multiple Factors	Total
None - found no effect	9	10	23	9	51
Negative effect	8	48	29	38	123
Positive effect	7	0	12	9	28
Both negative and positive	2	2	2	12	18
Total	26	60	66	68	220

```
Pearson's Chi-squared test

data:  bioenergyFFByEffect
X-squared = 44.829, df = 9, p-value = 9.924e-07
```

Supplementary Table 11: Chi-Squared expected cell counts if there is no relationship between food factors and overall effect on food security.

	Availability	Price	Production	Multiple Factors
None - found no effect	6.027273	13.909091	15.3	15.763636
Negative effect	14.536364	33.545454	36.9	38.018182
Positive effect	3.309091	7.636364	8.4	8.654545
Both negative and positive	2.127273	4.909091	5.4	5.563636

Again there are a few combinations with a small number of expected journal articles based on marginal totals, primarily in the “Both positive and negative” overall effect category and in the “Price” food factors

category. Again, a non-parametric Chi-Squared test of independence will be used to test for the relationship between food factors and the overall effect on the food security.

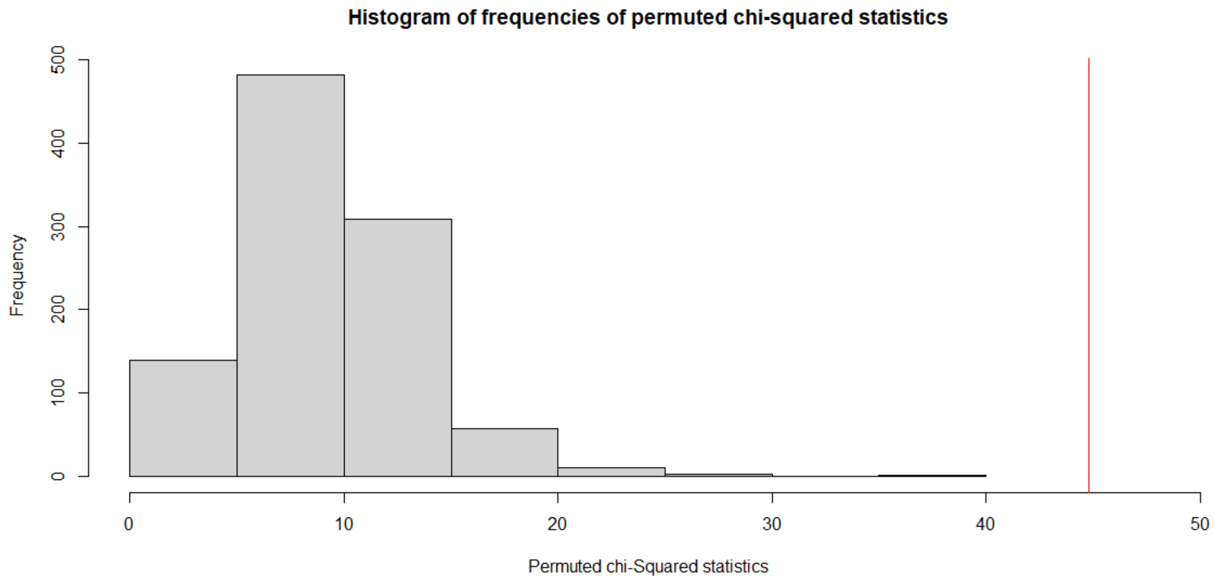

Supplementary Figure 8: Histogram of permuted test statistics for the Non-Parametric Chi-Squared test of a relationship between food factors and overall effect on food security. The vertical red line marks the observed Chi-Squared test statistic.

Both the parametric and non-parametric Chi-Squared tests indicate that there is strong evidence against there being no relationship between food factors and the overall effect on food security mentioned in the journal articles ($\chi^2 = 44.8289$, permutation-based p-value < 0.001).

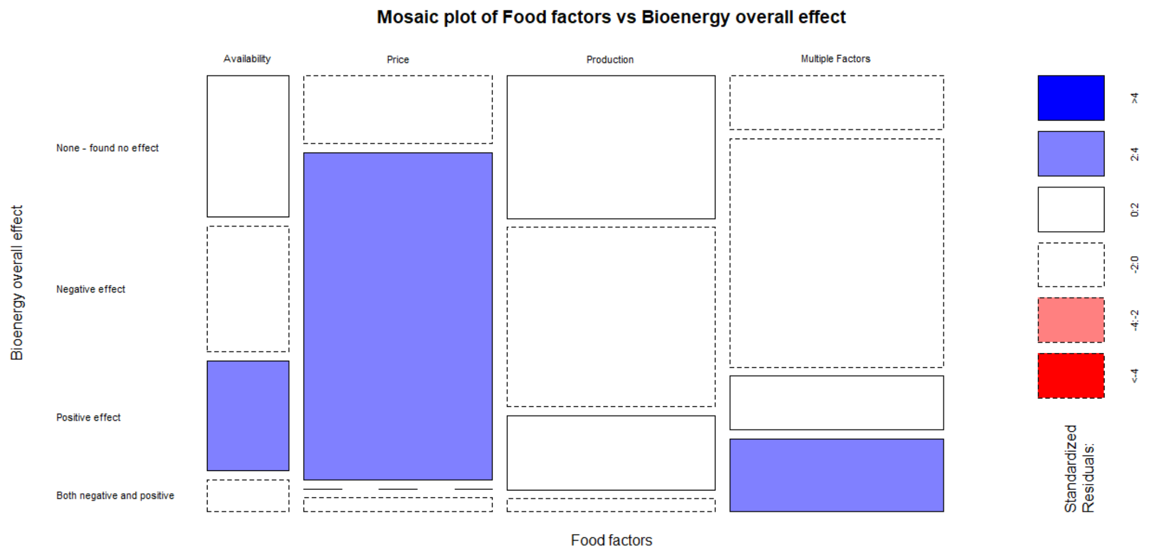

Supplementary Figure 9: Mosaic plot of standardized residuals for the food factors by overall effect model. Cells that are shaded light blue indicate there are three standardized residuals that are between 2 and 4 which is larger than would be expected if there was no relationship between food factors and overall effect. Cells with solid borders indicate positive residuals whereas cells with dashed borders indicate negative residuals.

The mosaic plot above indicates that there are three combinations with a standardized residual that is larger than would be expected if there was no relationship between the food factors and the overall effect on food security. The combinations include food price and negative effect, food availability and positive effect, and multiple factors and both positive and negative effects. This seems to indicate that if a paper indicated both positive and negative effects, the paper also likely considered multiple food factors. Also there were more papers than would be expected that indicated the overall effect was negative that also considered food price as a factor. Lastly there were more papers than would be expected that found an overall positive effect that considered food availability as a factor. The rest of the combinations were about what you would expect if there was no relationship between food factors and overall effect on food security.

Next, we analyzed overall effect by socio-demographic index (Low, Low-Middle, Middle, High-Middle, High, Multiple, Global SDI) group.

Supplementary Table 12: The number of journal articles by SDI group and overall effect on food security.

	Low	Low-Middle	Middle	High-Middle	High	Multiple SDI	Global SDI	Total
None - found no effect	3	4	7	6	15	6	10	51
Negative effect	4	7	12	8	36	14	42	123
Positive effect	10	1	6	1	6	2	2	28
Both negative and positive	4	1	5	1	3	1	3	18
Total	21	13	30	16	60	23	57	220

```
    Pearson's Chi-squared test

data:  bioenergySDIByEffect
X-squared = 47.547, df = 18, p-value = 0.0001758
```

Supplementary Table 13: Chi-Squared expected cell counts if there is no relationship between SDI group and overall effect on food security.

	Low	Low-Middle	Middle	High-Middle	High	Multiple SDI	Global SDI
None - found no effect	4.868182	3.013636	6.954546	3.709091	13.909091	5.331818	13.213636
Negative effect	11.740909	7.268182	16.772727	8.945455	33.545454	12.859091	31.868182
Positive effect	2.672727	1.654546	3.818182	2.036364	7.636364	2.927273	7.254545
Both negative and positive	1.718182	1.063636	2.454546	1.309091	4.909091	1.881818	4.663636

There are a few combinations with a small number of expected journal articles based on marginal totals, primarily in the “Both positive and negative” and “Positive effect” overall effect categories as well as in the “None - found now effect” category. Every SDI group has at least one cell with a small expected number of journal articles. Again, a non-parametric Chi-Squared test of independence will be used to test for the relationship between SDI groups and the overall effect on the food security.

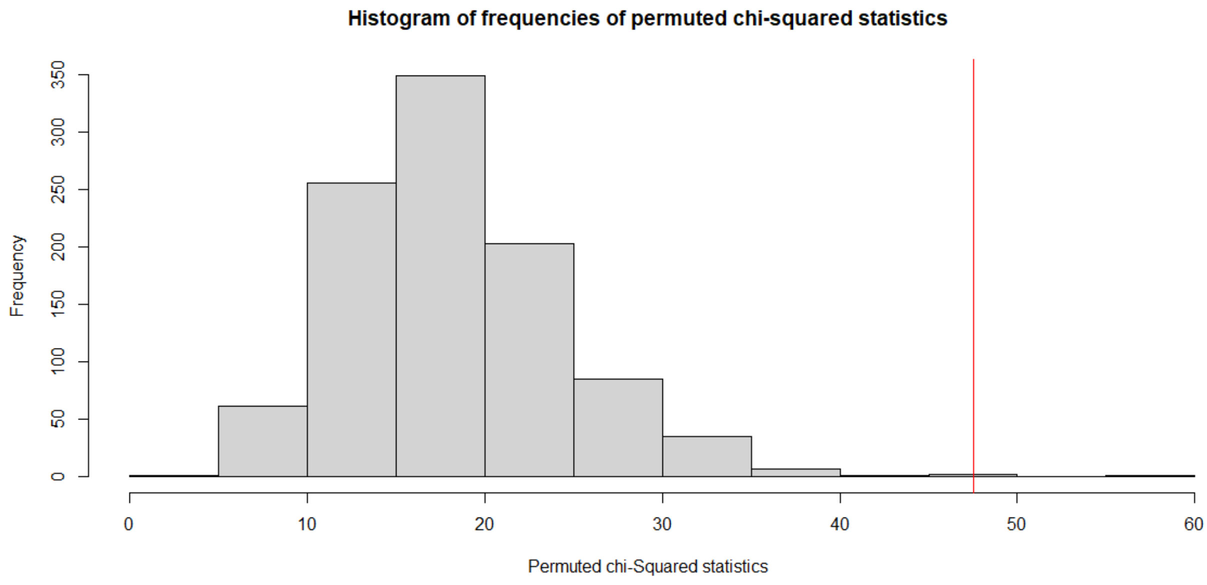

Supplementary Figure 10: Histogram of permuted test statistics for the Non-Parametric Chi-Squared test of a relationship between SDI group and overall effect on food security. The vertical red line marks the observed Chi-Squared test statistic.

Both the parametric and non-parametric Chi-Squared tests indicate that there is strong evidence against there being no relationship between SDI groups and the overall effect on food security mentioned in the journal articles ($\chi^2 = 47.5466$, permutation-based p-value = 0.001).

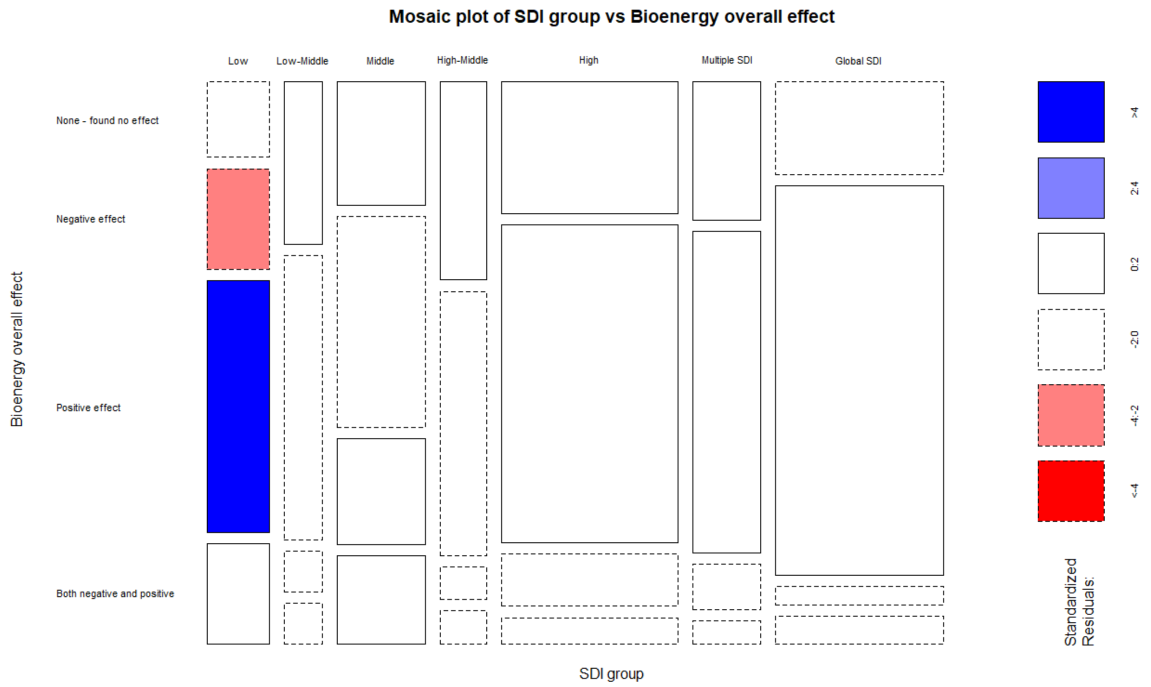

Supplementary Figure 11: Mosaic plot of standardized residuals for the SDI group by overall effect model. The cell that is shaded dark blue indicate there are is one standardized residual that is greater than 4 which is much larger than would be expected if there was no relationship between SDI group and overall effect. One cell is shaded light red, meaning its standardized residual is between -4 and -2 and is smaller than would be expected if there was no relationship between SDI group and overall effect. Cells with solid borders indicate positive residuals whereas cells with dashed borders indicate negative residuals.

From the mosaic plot we can see combinations contributing the most to the Chi-Squared statistic. The standardized residual for the low SDI group and positive overall effect is much larger than would be expected if there was no relationship between SDI group and overall effect on food security. The residual for low-middle SDI and Negative effect is smaller than would be expected, meaning there were fewer papers reporting a negative effect for low-Middle SDI countries than would be expected if there was no relationship between SDI group and overall effect on food security.

Then, we analyzed overall effect by spatial scale (Household, Community, Regional, National, Multinational, Global, and Multiple scales).

Supplementary Table 14: The number of journal articles by spatial scale and overall effect on food security.

	Household	Community	Regional	National	Multinational	Global scale	Multiple scales	Total
None - found no effect	3	2	11	20	5	10	0	51
Negative effect	2	9	18	38	11	43	2	123
Positive effect	8	1	9	6	1	2	1	28
Both negative and positive	1	0	4	9	1	3	0	18
Total	14	12	42	73	18	58	3	220

Pearson's Chi-squared test

data: bioenergySpatByEffect

X-squared = 47.76, df = 18, p-value = 0.0001635

Supplementary Table 15: Chi-Squared expected cell counts if there is no relationship between spatial scale and overall effect on food security.

	Household	Community	Regional	National	Multi-national	Global scale	Multiple scales
None - found no effect	3.245455	2.7818182	9.736364	16.922727	4.172727	13.445455	0.6954545
Negative effect	7.827273	6.7090909	23.481818	40.813636	10.063636	32.427273	1.6772727
Positive effect	1.781818	1.5272727	5.345454	9.290909	2.290909	7.381818	0.3818182
Both negative and positive	1.145455	0.9818182	3.436364	5.972727	1.472727	4.745455	0.2454545

There are many combinations with a small number of expected journal articles based on marginal totals, primarily in the “Both positive and negative” and “Positive effect” overall effect categories. Again, a non-parametric Chi-Squared test of independence will be used to test for the relationship between spatial scale and the overall effect on the food security.

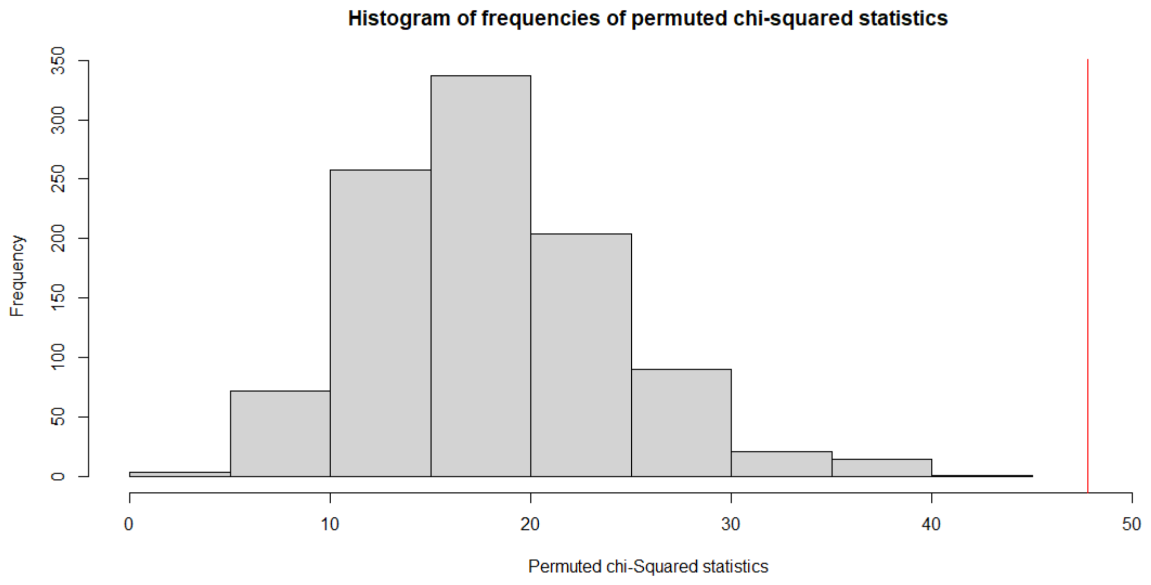

Supplementary Figure 12: Histogram of permuted test statistics for the Non-Parametric Chi-Squared test of a relationship between spatial scale and overall effect on food security. The vertical red line marks the observed Chi-Squared test statistic.

Both the parametric and non-parametric Chi-Squared tests indicate that there is strong evidence against there being no relationship between spatial scale and the overall effect on food security mentioned in the journal articles ($\chi^2 = 47.7596$, permutation-based p-value < 0.001).

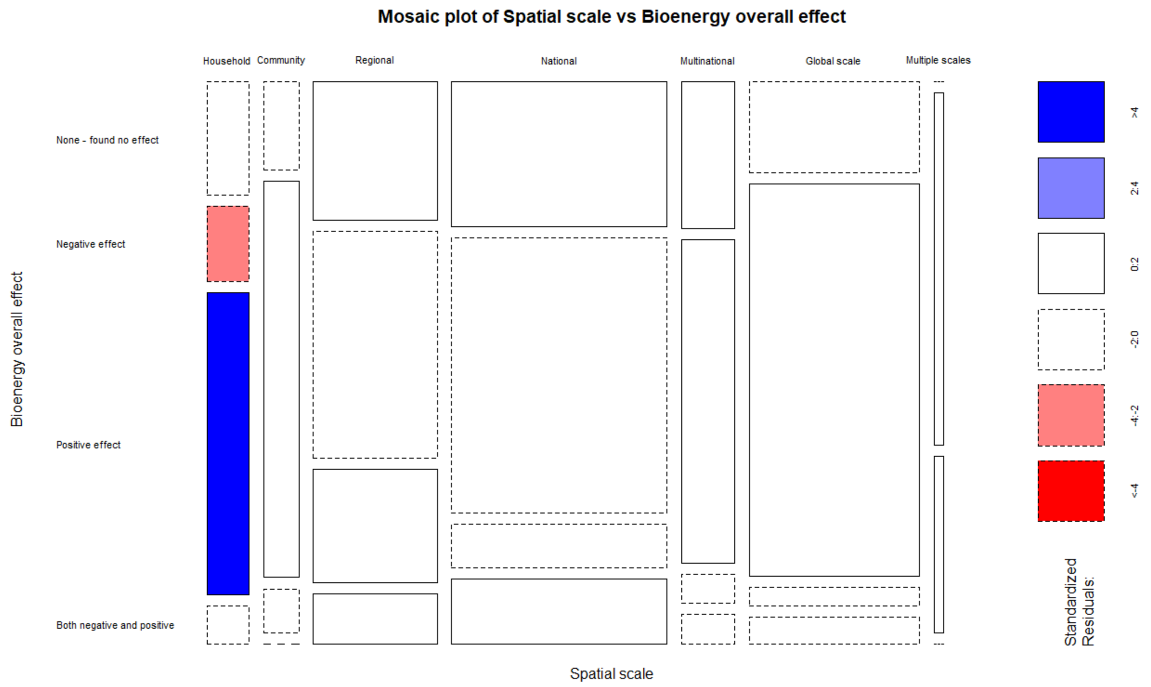

Supplementary Figure 13: Mosaic plot of standardized residuals for the spatial scale by overall effect. The cell that is shaded dark blue indicate there are is one standardized residual that is greater than 4 which is much larger than would be expected if there was no relationship between spatial scale and overall effect. One cell is shaded light red, meaning its standardized residual is between -4 and -2 and is smaller than would be expected if there was no relationship between spatial scale and overall effect. Cells with solid borders indicate positive residuals whereas cells with dashed borders indicate negative residuals.

From the mosaic plot we can see combinations contributing the most to the Chi-Squared statistic. The standardized residual for the household scale and positive overall effect is much larger than would be expected if there was no relationship between spatial scale and overall effect on food security. The residual for household and negative effect is smaller than would be expected, meaning there were fewer papers reporting a negative effect for household countries than would be expected if there was no relationship between spatial scale and overall effect on food security.

Next, we analyzed overall effect by temporal scale (Short-term, Long-term, Both short,- and long-term, and Term not specified).

Supplementary Table 16: The number of journal articles by temporal scale and overall effect on food security.

	Term not specified	Short term	Long term	Both terms	Total
None - found no effect	2	16	31	2	51
Negative effect	9	21	88	5	123
Positive effect	0	14	14	0	28
Both negative and positive	2	2	13	1	18
Total	13	53	146	8	220

```
	Pearson's Chi-squared test

data:  bioenergyTempByEffect
X-squared = 19.164, df = 9, p-value = 0.02383
```

Supplementary Table 17: Chi-Squared expected cell counts if there is no relationship between temporal scale and overall effect on food security.

	Term not specified	Short term	Long term	Both terms
None - found no effect	3.013636	12.286364	33.84545	1.8545455
Negative effect	7.268182	29.631818	81.62727	4.4727273
Positive effect	1.654546	6.745455	18.58182	1.0181818
Both negative and positive	1.063636	4.336364	11.94545	0.6545455

There are a few combinations with a small number of expected journal articles based on marginal totals, primarily in the “Both positive and negative” and “Positive effect” overall effect categories. Again, a non-parametric Chi-Squared test of independence will be used to test for the relationship between temporal scale and the overall effect on the food security.

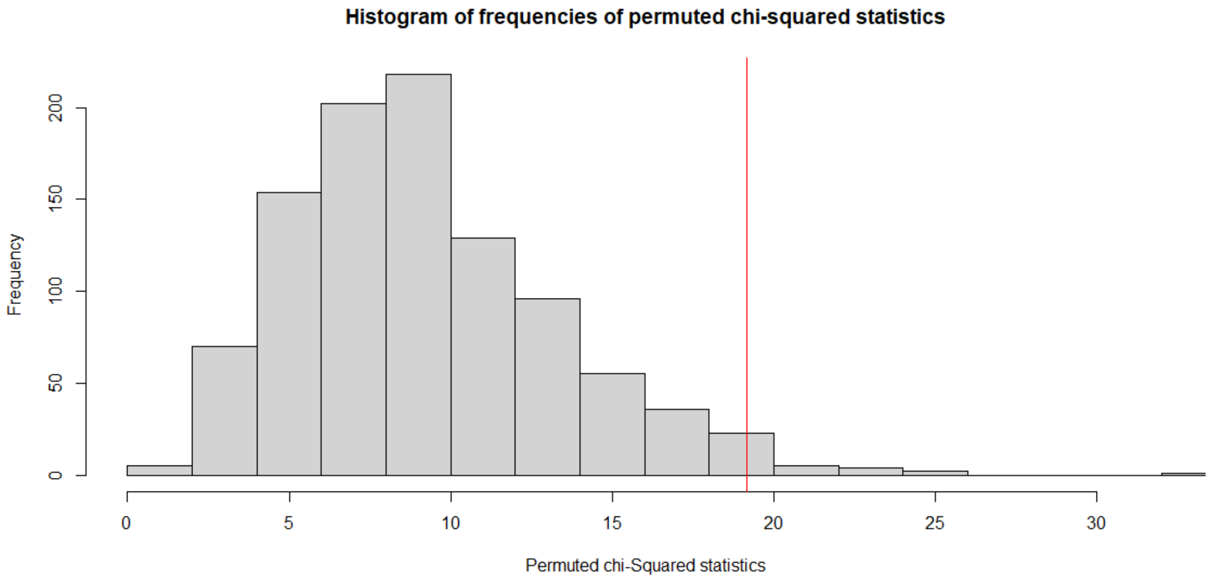

Supplementary Figure 14: Histogram of permuted test statistics for the Non-Parametric Chi-Squared test of a relationship between temporal scale and overall effect on food security. The vertical red line marks the observed Chi-Squared test statistic.

Both the parametric and non-parametric Chi-Squared tests indicate that there is moderate evidence against there being no relationship between temporal scale and the overall effect on food security mentioned in the journal articles ($\chi^2 = 19.1639$, permutation-based p-value = 0.017).

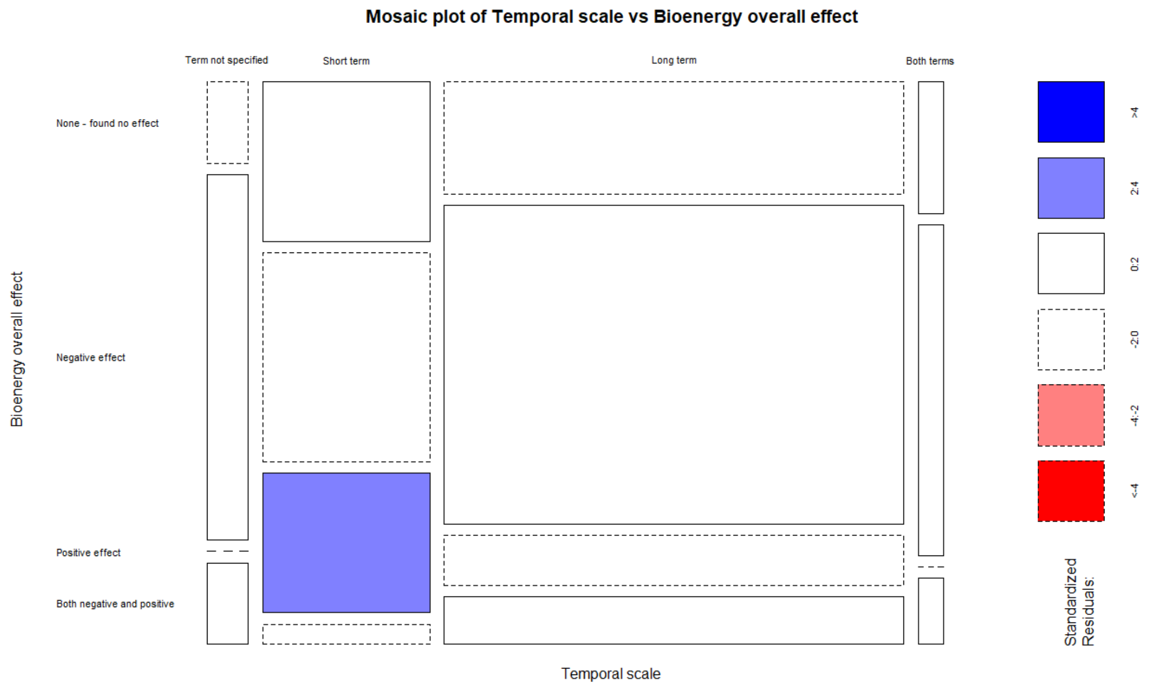

Supplementary Figure 15: Mosaic plot of standardized residuals for the temporal scale by overall effect. The cell that is shaded light blue indicate there are is one standardized residual that is between 2 and 4 which is larger than would be expected if there was no relationship between temporal scale and overall effect. Cells with solid borders indicate positive residuals whereas cells with dashed borders indicate negative residuals.

From the mosaic plot we can see combinations contributing the most to the Chi-Squared statistic. The standardized residual for the short term and positive overall effect is much larger than would be expected if there was no relationship between temporal scale and overall effect on food security.

Next, we analyzed overall effect by dataset type (Observed, Predicted, and Both Observed and Predicted).

Supplementary Table 18: The number of journal articles by dataset type and overall effect on food security.

	Both	Observed	Predicted	Total
None - found no effect	6	25	20	51
Negative effect	11	49	63	123
Positive effect	0	19	9	28
Both negative and positive	0	6	12	18
Total	17	99	104	220

```
	Pearson's Chi-squared test

data:  bioenergyDTByEffect
X-squared = 13.466, df = 6, p-value = 0.0362
```

Supplementary Table 19: Chi-Squared expected cell counts if there is no relationship between dataset type and overall effect on food security.

	Both	Observed	Predicted
None - found no effect	3.940909	22.95	24.109091
Negative effect	9.504546	55.35	58.145454
Positive effect	2.163636	12.60	13.236364
Both negative and positive	1.390909	8.10	8.509091

There are three combinations with a small number of expected journal articles based on marginal totals, all in the “Both” category for the dataset type. Again, a non-parametric Chi-Squared test of independence will be used to test for the relationship between spatial scale and the overall effect on the food security.

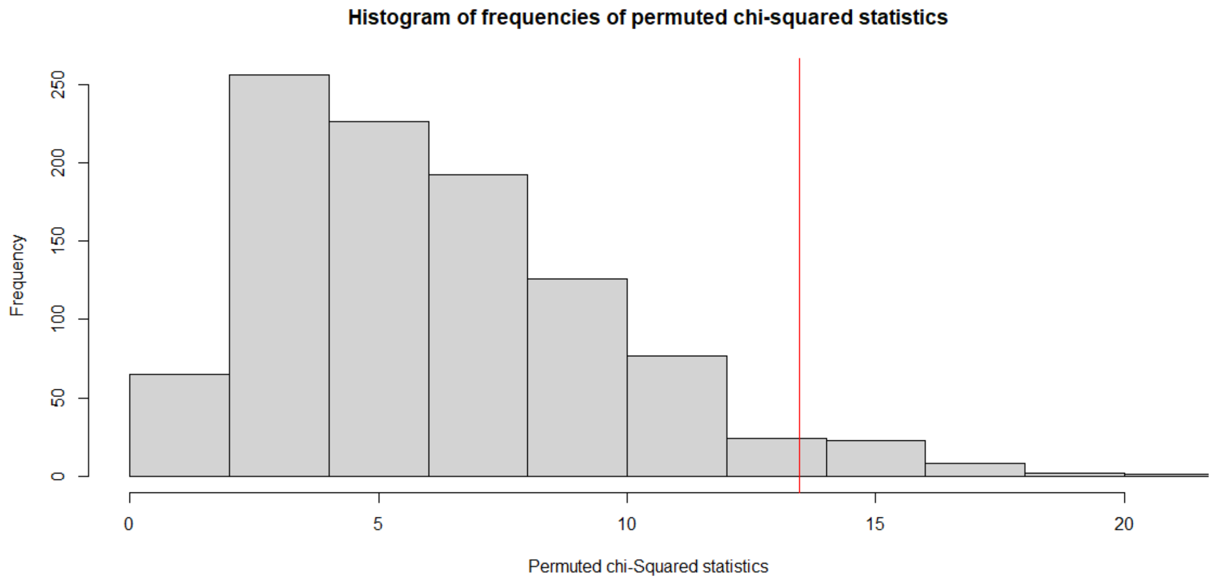

Supplementary Figure 16: Histogram of permuted test statistics for the Non-Parametric Chi-Squared test of a relationship between dataset and overall effect on food security. The vertical red line marks the observed Chi-Squared test statistic.

Both the parametric and non-parametric Chi-Squared tests indicate that there is moderate evidence against there being no relationship between dataset and the overall effect on food security mentioned in the journal articles ($\chi^2 = 13.4662$, permutation-based p-value = 0.036).

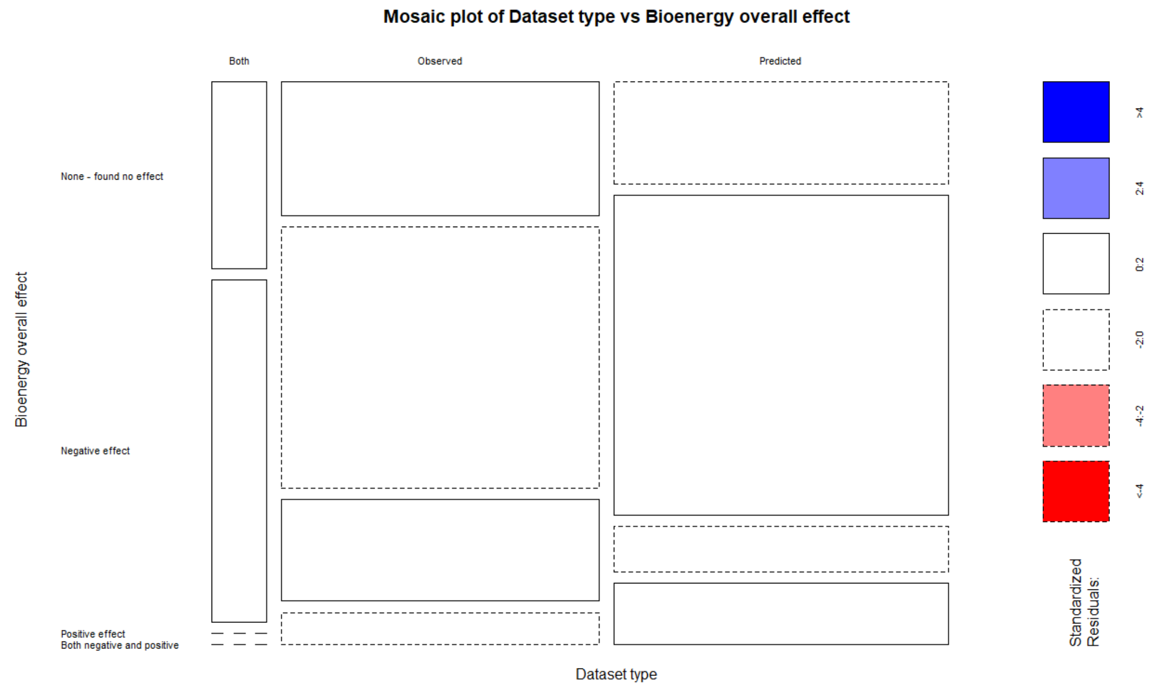

Supplementary Figure 17: Mosaic plot of standardized residuals for the dataset type by overall effect. No standardized residuals are noticeably large in this result. Cells with solid borders indicate positive residuals whereas cells with dashed borders indicate negative residuals.

In this situation, there are no cells that provide a clear contribution to the test statistic that found moderate evidence of a relationship between data set type and bioenergy overall effect. It is interesting that the "observed" data sets had fewer negative effects than expected and the "predicted" ones had more than if no relationship were present, but the sizes of the differences from expected are not that large.

Statistical learning methods provide a way to explore papers for common characteristics from the multivariate information available using dimension reduction and clustering techniques to visualize and identify common patterns among the papers. These explorations seek to identify common patterns on bioenergy feedstock, food security parameter, temporal scale, spatial scale, and SDI group (see alluvial diagram in Supplementary Figure 21 for details on levels) and then explore potential connections of those identified clusters with the overall effect identified in the papers. This analysis excludes four papers that

did not clearly specific Edible or Inedible bioenergy feedstock, so used n=220 observations. Gower's dissimilarity (Gower (1971)) is used to compare the multivariate categorical aspects of the papers using a weighted combination of the differences on each variable. In the calculation of Gower's dissimilarity, papers in the same category on a variable are measured as a difference of 0 and different categories (any combination) treated at 1, and these are averaged across all the variables to create the overall dissimilarity measure. Three techniques are used to explore connections among the papers. First, papers are clustered using hierarchical cluster analysis with Ward's method (Murtagh and Legendre (2014)), then the clusters are visualized in a two-dimensional map using multi-dimensional scaling with minimum spanning trees (Oksanen et al. (2019)) (minimum spanning trees show connections among closest neighboring papers), and finally alluvial diagrams are used to explore connections of the cluster identifiers and the features used to create them to more fully characterize the clusters and explore links between them and the type of overall effect found in the papers. Note that the type of overall effect was not used in the dimension reduction or cluster analysis so that the clusters could be compared to that characteristic.

Cluster analysis provides a tool for identifying groups of manuscripts with similar characteristics within a cluster and different ones among the clusters (see Everitt et al. (2011)). Hierarchical cluster analysis depends on the choice of dissimilarity (Gower's), the agglomeration method which we chose as Ward's linkage method (Murtagh and Legendre (2014)) as it created more distinct clusters with these data than some other linkage methods we explored, and the choice of the number of clusters. Based on the heights of mergers in the dendrogram, there appear to be four distinct clusters. Given the results for the four clusters, we can proceed to try to understand the characteristics of those clusters, by using the two-dimensional map presented in Supplementary Figure 18 and in an alluvial diagram (Supplementary Figure 21). Note that each of the choices can have a dramatic impact on the cluster analysis solution and interpretation.

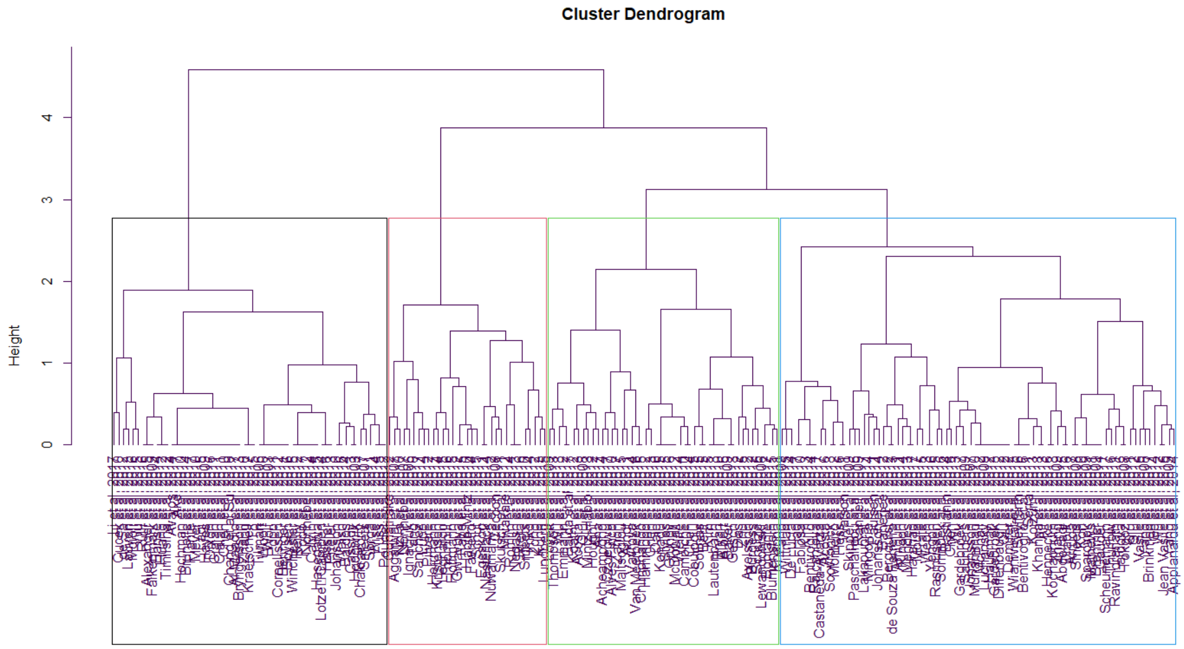

Supplementary Figure 18: Dendrogram of hierarchical cluster analysis based on Gower's dissimilarity and Ward's method, with four selected clusters highlighted.

Classical multi-dimensional scaling is a dimension reduction technique for visualizing similarities/differences among observations in a map based on the relative proximities of the papers - here that is based on bioenergyFeedstock, foodFactors, temporalScale, spatialScale, and SDI information that is also used in the cluster analysis. The Euclidean distance in the plots provides information on the relative close-ness/far-ness of the papers which is created based on the Gower's dissimilarity matrix. There is no clear interpretation of the axis directions as equivalent maps can be rotated, flipped, and re-scaled. The lines between papers are based on minimum spanning trees that link close neighbors in the original Gower's dissimilarities. These linkages are useful for identifying possible close neighbors in response profiles and for looking for distortions in making the two-dimensional map from the higher dimensional dissimilarity data. Supplementary Figure 19 displays the map of papers with their coloring and neighbor linkages based on the four cluster solution. This plot reinforces that relatively clear spacing

of the clusters that were found in the dendrogram, with cluster 1 (purple) in the lower left corner of the plot (with a few exceptions), cluster 2 (blue) in the right part of the plot, cluster 3 (teal) in the center of the plot, and cluster 4 (green) in the upper left of the plot. There are exceptions to this description and those papers might be less clearly linked to the papers in their cluster - or placed in a location that is a distortion of the overall dissimilarities due to plotting the high dimensional space in two dimensions.

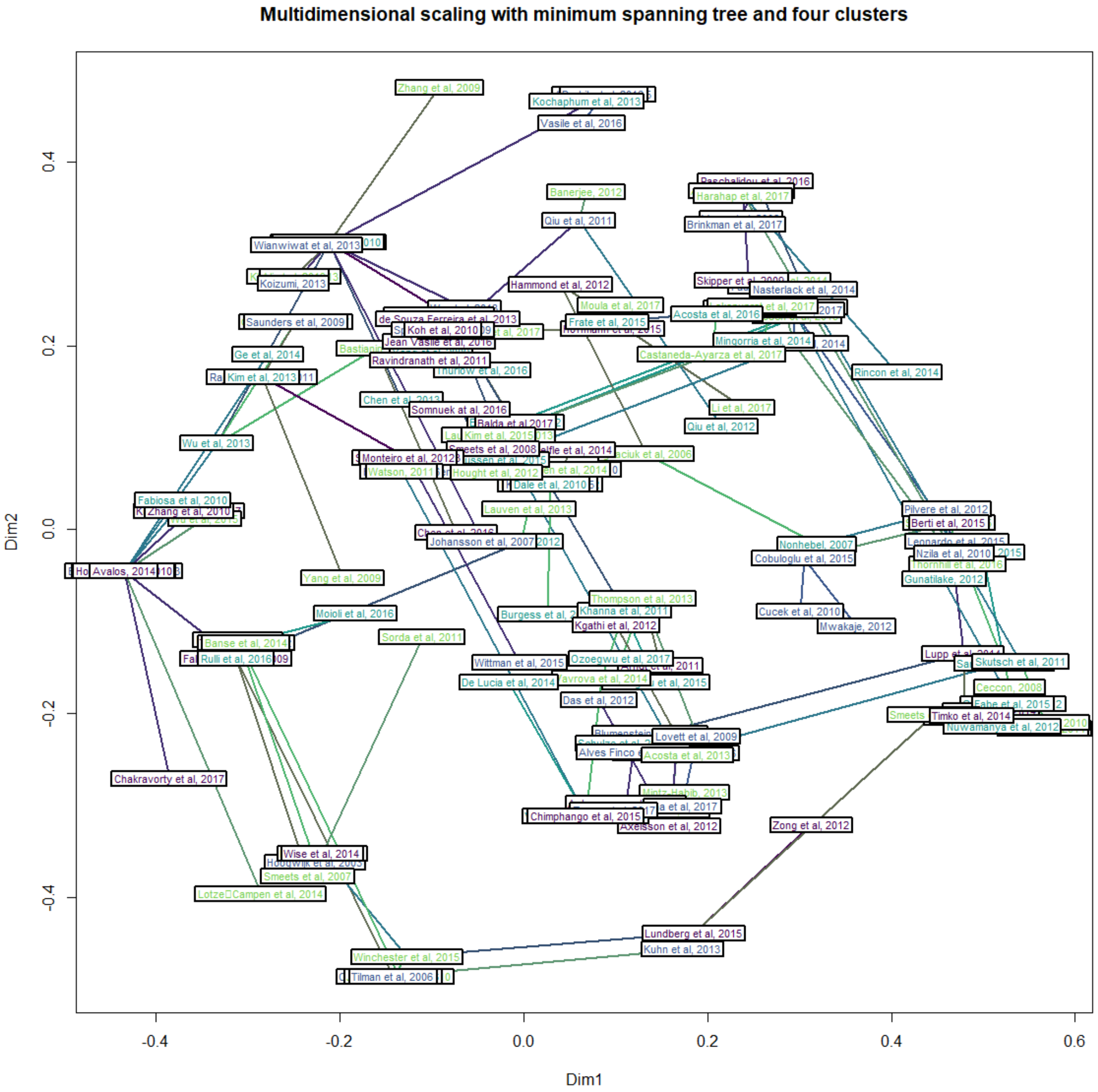

Supplementary Figure 19: Dendrogram of hierarchical cluster analysis based on Gower's dissimilarity and Ward's method, with four selected clusters highlighted.

We can also compare the resulting clusters to the overall effect variable that was not used to develop the clusters to attempt to identify connections between the suite of characteristics used to create the clusters and the outcome of each article. Cluster 1 had the highest proportion of negative effects, cluster 2 had the higher proportion of positive effects and no effect found, cluster 3 had the highest proportion of both

types of effects, and was not unusual in any of its proportions but had many negative effect results. Each cluster had some of all types of effects and the differences across the clusters was not too dramatic.

	1	2	3	4
None - found no effect	11	12	13	15
Negative effect	41	9	22	51
Positive effect	2	10	8	8
Both negative and positive	3	2	5	8

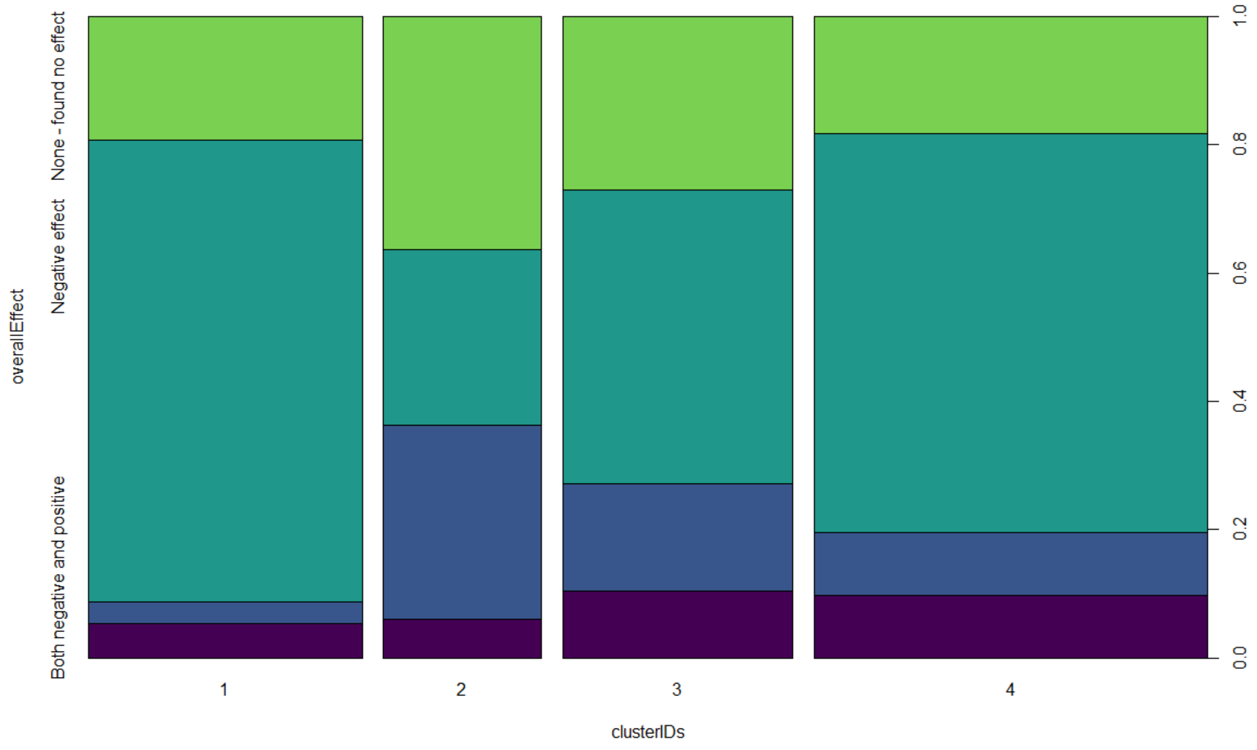

Supplementary Figure 20: Stacked bar chart of the overall effect category proportions in each cluster.

Supplementary Figure 21 shows an alluvial diagram of the cluster membership for the four cluster solution and how those clusters relate to the variables used to create the clusters (bioenergy feedstock, food security parameter, temporal scale, spatial scale, and SDI group) followed by the effect found.

Tracing the flows of the clusters across the variables provides potential interpretations of each cluster. Cluster 1 (purple) has a large proportion of Edible bioenergy that also tracks into Price for the food factors and long term time scale and global scale and global SDI that also relate a large proportion of negative effects found. Cluster 2 (blue) includes a larger proportion of Inedible bioenergy with notable proportions of either Availability or Production food factors, with no term specified for the time scale, household and community spatial scale, mixed SDI levels and types of effects - with no clear group dominating for either of those variables. Cluster 3 (teal) has nearly equal proportions of Edible, Inedible, and both types of bioenergy, that mainly contain production or multiple food factors with long term time scales and regional and national spatial scale, a mix of SDI scales with more of the higher levels and a mix of different types of effects. Cluster 4 (green) is mainly Edible bioenergy with large proportions in price, production, and multiple factors for food factors, short or long term temporal scales with mostly national or multinational scales and mix of SDI levels. Cluster 4 had a high proportion of negative effect and then no effect outcomes.

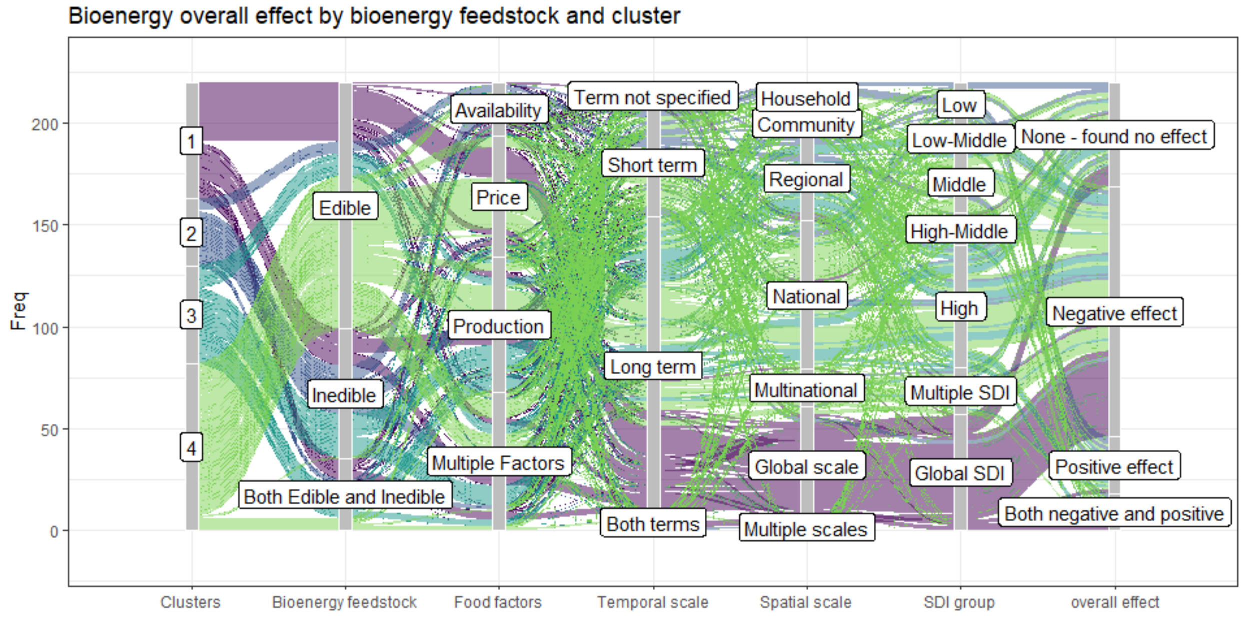

Supplementary Figure 21: Alluvial plot of the bioenergy feedstock, the food factors and the overall effect of the bioenergy on food security from a review of 220 journal articles, with clusters.

This following section describes a machine learning approach to uncover the effects of bioenergy on food security from the published studies described above. The use of machine learning is robust to the relatively small sample size of the present study and can account for the very large number of interactions between paper-level variables that need to be accounted for to treat the full scope of the relationships. Natural language processing techniques (e.g. TFIDF, non-negative matrix factorization to project the text into a metric space) on text-heavy variables were explored but were adjudged to be unsuitable due to limitations in the vocabulary within these variables. Likewise, a maximum likelihood estimation approach was rejected for two reasons. First, the dataset is relatively small and each sub-dataset is smaller yet, and relying on the asymptotic properties of frequentist hypothesis testing with these data seemed questionable. Second, the explanatory variable effects are likely dependent on interactions between them. This implies a potentially large number of terms to include in the model: 63 interactive terms to capture all interactions in the original six variables and more than 1 million for an exhaustive treatment of the binary indicators. This would also have complicated post-analysis interpretation.

The methods used to conduct a study likely depends on the dependent variable studied. To account for this, three models were tested for the three major categories of dependent variables found within the papers in the sample: food price, food production, and food availability. By separating data into subsets that focused on only a single type of dependent variable (price, production, availability) the problem of modeling the dependent variable became a straightforward multi-categorial problem. Six explanatory variables were included in each of the models: SDI, bioenergy type, bioenergy generation, spatial scale, temporal scale, and data type. Data were transformed into multiple binary indicators, one for each category in the domain of the variable.

The relationships between overall effect and the paper-level variables was extracted using a machine learning approach. Random forest models are decision-tree ensembles in which each tree is constructed via bootstrap aggregation and sampling from the explanatory variable space (Breiman (2001)). They are suitable for capturing complex interactions between explanatory variables. The impact of each binary

indictor created from the original six variables was assessed via partial dependence plots (Friedman (2001)).

The study-level variables on the found effects from the sample of papers reveals different effects across the three food security categories. This suggests that global conclusions about the effect of bioenergy on food security are not warranted and that effects depend on the specific aspect of food security examined. The following summary focuses on effects that are robust in the sense they are obtained irrespective of the other variables they interact with.

In modeling the effect of study-level variables on price, studies that focused on edible biofuels or that took a global view were more likely to find a negative effect of bioenergy production (Supplementary Figure 22). Those that included middle SDI countries were more likely to lead to ambiguous effects with nearly all combinations of study-level variables resulting in clear increases in the probability of either both positive and negative effects. Finally, price effects derived from bioenergy are difficult to detect by focusing on low SDI countries, or by studying the household or regional level – inclusion of either of these was associated with finding no effect.

The papers that included low SDI countries or that analyzed the household level were more likely to find positive effects of bioenergy on production (Supplementary Figure 23). In terms of food availability, those papers that included household level analysis were more likely to find positive effects of bioenergy on food security while those that included low-middle SDI countries, that focused their work at the community level, or that studied first generation biofuels were more likely to find negative effects (Supplementary Figure 24).

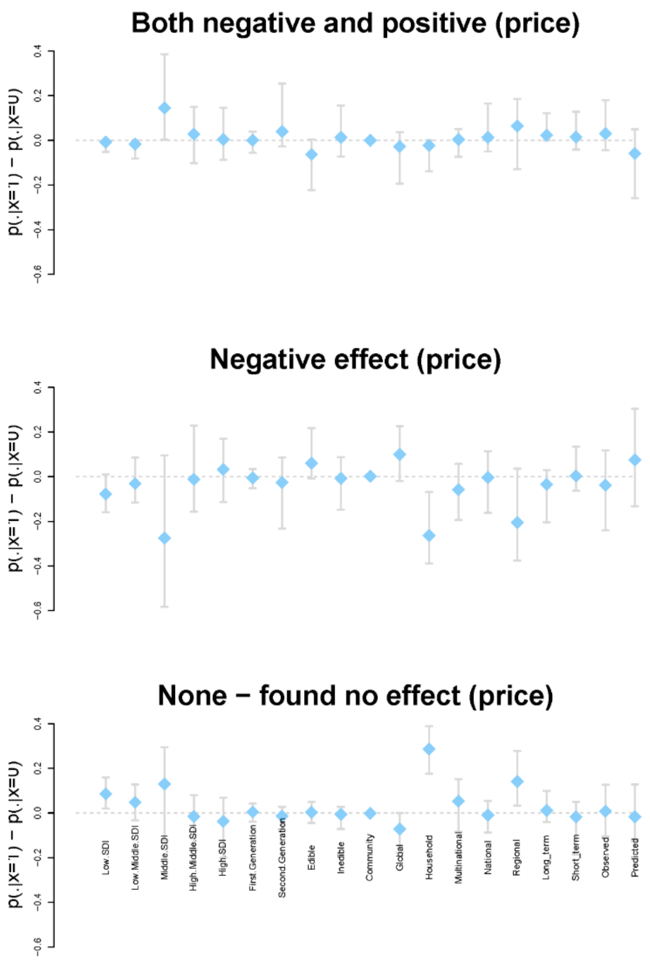

Supplementary Figure 22: Partial dependence plots of included variable effects on Overall Effect for papers that studied food price. All variables are binary indicators. The y-axis measures the change in probability of each category for inclusion of the variable. The vertical lines represent the empirical 95 percent confidence interval over the change in probability across the different observations in the training data and the points plotted are the average.

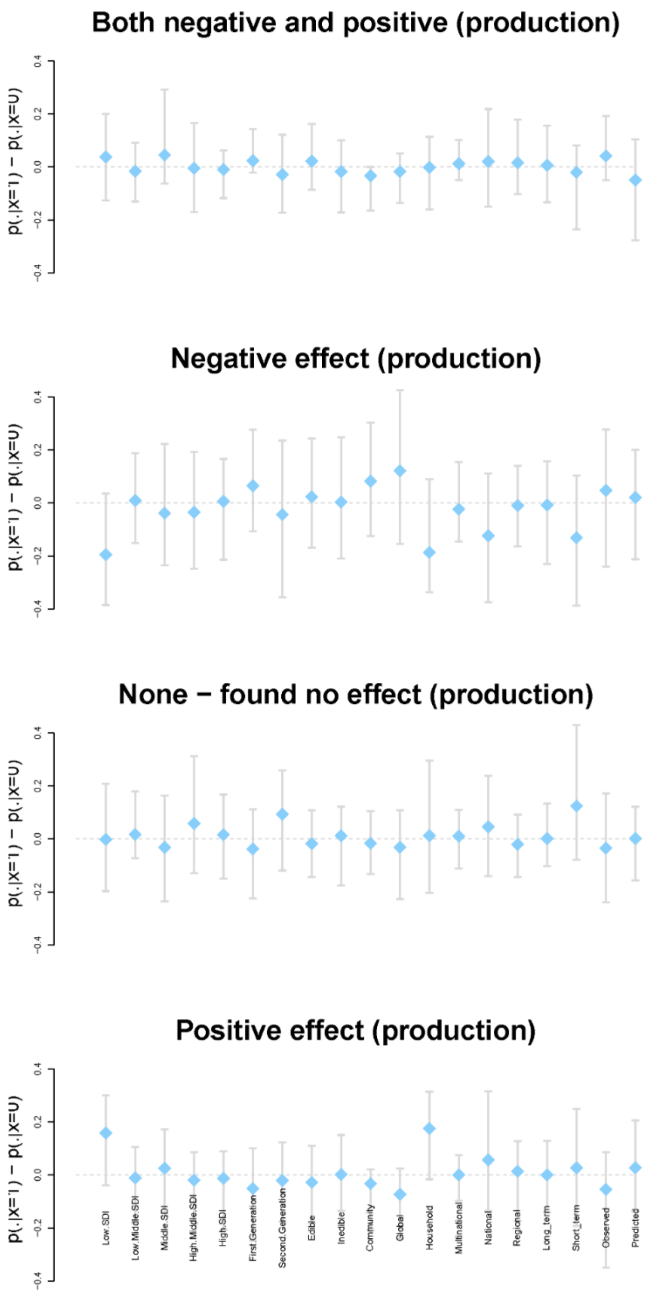

Supplementary Figure 23: Partial dependence plots of included variable effects on Overall Effect for papers that studied food production. All variables are binary indicators. The y-axis measures the change in probability of each category for inclusion of the variable. The vertical lines represent the empirical 95 percent confidence interval over the change in probability across the different observations in the training data and the points plotted are the average.

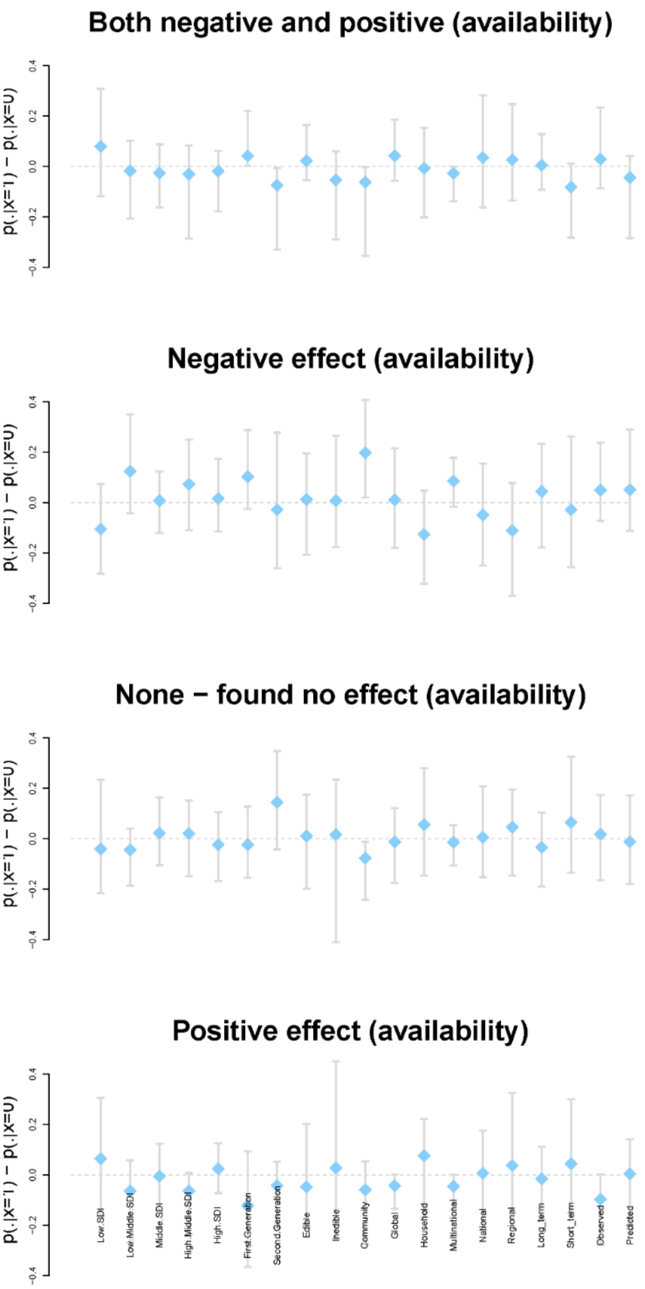

Supplementary Figure 24: Partial dependence plots of included variable effects on Overall Effect for papers that studied food availability. All variables are binary indicators. The y-axis measures the change in probability of each category for inclusion of the variable. The vertical lines represent the empirical 95 percent confidence interval over the change in probability across the different observations in the training data and the points plotted are the average.

The R package bookdown (Xie 2020a) was used to create this report document using the R language (R Core Team 2020). In addition, the following were packages used for the analysis and/or formatting of this document:

- bookdown (Xie 2020a)
- knitr (Xie 2020b)
- rmarkdown (Allaire et al. 2020)
- readxl (Wickham and Bryan 2019)
- tibble (Müller and Wickham 2020)
- dplyr (Wickham et al. 2020)
- tidyr (Wickham 2020)
- mosaic (Pruim, Kaplan, and Horton 2020)
- effects (Fox et al. 2019)
- ggalluvial (Brunson 2020)
- nnet (Ripley 2020)
- car (Fox, Weisberg, and Price 2020)
- cluster (Maechler et al. 2019)
- vegan (Oksanen et al. 2019)
- circlize (Gu 2020)
- RColorBrewer (Neuwirth 2014)
- viridis (Garnier 2018)

Supplementary References

Allaire, JJ, Yihui Xie, Jonathan McPherson, Javier Luraschi, Kevin Ushey, Aron Atkins, Hadley Wickham, Joe Cheng, Winston Chang, and Richard Iannone. 2020. *Rmarkdown: Dynamic Documents for R*. https://CRAN.R-project.org/package=rmarkdown.

Brunson, Jason Cory. 2020. *Ggalluvial: Alluvial Plots in 'Ggplot2'*. https://CRAN.R-project.org/package=ggalluvial.

Breiman, L. *Random Forests*. 2001. Accessed January 7, 2021 https://www.stat.berkeley.edu/~breiman/randomforest2001.pdf

Everitt, Brian S., Sabine Landau, Morven Leese, and Daniel Stahl. 2011. *Cluster Analysis*. 5th ed. Wiley.

Fox, John, Sanford Weisberg, and Brad Price. 2020. *Car: Companion to Applied Regression*. https://CRAN.R-project.org/package=car.

Fox, John, Sanford Weisberg, Brad Price, Michael Friendly, and Jangman Hong. 2019. *Effects: Effect Displays for Linear, Generalized Linear, and Other Models*. https://CRAN.R-project.org/package=effects.

Friedman, J.H., 2001. Greedy function approximation: A gradient boosting machine. Ann. Statist. 29, 1189–1232. https://doi.org/10.1214/aos/1013203451

Garnier, Simon. 2018. *Viridis: Default Color Maps from 'Matplotlib'*. https://CRAN.R-project.org/package=viridis.

Gower, J. C. 1971. "A General Coefficient of Similarity and Some of Its Properties." *Biometrics* 27 (4): 857–71. http://www.jstor.org/stable/2528823.

Greenwood, Mark. 2020. *Intermediate Statistics with R* (version 2.2). https://doi.org/https://doi.org/10.15788/2019.08.19.

Gu, Zuguang. 2020. *Circlize: Circular Visualization*. https://CRAN.R-project.org/package=circlize.

Maechler, Martin, Peter Rousseeuw, Anja Struyf, and Mia Hubert. 2019. *Cluster: "Finding Groups in Data": Cluster Analysis Extended Rousseeuw et Al.* https://CRAN.R-project.org/package=cluster.

Murtagh, Fionn, and Pierre Legendre. 2014. "Ward's Hierarchical Agglomerative Clustering Method: Which Algorithms Implement Ward's Criterion?" *Journal of Classification* 31 (3): 274–95. https://doi.org/https://doi.org/10.1007/s00357-014-9161-z.

Müller, Kirill, and Hadley Wickham. 2020. *Tibble: Simple Data Frames*. https://CRAN.R-project.org/package=tibble.

Neuwirth, Erich. 2014. *RColorBrewer: ColorBrewer Palettes*. https://CRAN.R-project.org/package=RColorBrewer.

Oksanen, Jari, F. Guillaume Blanchet, Michael Friendly, Roeland Kindt, Pierre Legendre, Dan McGlinn, Peter R. Minchin, et al. 2019. *Vegan: Community Ecology Package*. https://CRAN.R-project.org/package=vegan.

Pruim, Randall, Daniel T. Kaplan, and Nicholas J. Horton. 2020. *Mosaic: Project Mosaic Statistics and Mathematics Teaching Utilities*. https://CRAN.R-project.org/package=mosaic.

R Core Team. 2020. *R: A Language and Environment for Statistical Computing*. Vienna, Austria: R Foundation for Statistical Computing. https://www.R-project.org/.

Ripley, Brian. 2020. *Nnet: Feed-Forward Neural Networks and Multinomial Log-Linear Models*. https://CRAN.R-project.org/package=nnet.

Wickham, Hadley. 2020. *Tidyr: Tidy Messy Data*. https://CRAN.R-project.org/package=tidyr.

Wickham, Hadley, and Jennifer Bryan. 2019. *Readxl: Read Excel Files*. https://CRAN.R-project.org/package=readxl.

Wickham, Hadley, Romain François, Lionel Henry, and Kirill Müller. 2020. *Dplyr: A Grammar of Data Manipulation*. https://CRAN.R-project.org/package=dplyr.

Xie, Yihui. 2020a. *Bookdown: Authoring Books and Technical Documents with R Markdown*. https://CRAN.R-project.org/package=bookdown. 2020b. *Knitr: A General-Purpose Package for Dynamic Report Generation in R*. https://CRAN.R-project.org/package=knitr.

Supplementary Table 20. Search terms and number of publications for each database included in meta-analysis.

Search Terms			Database						
**Type of biofuel**		**Food Security Parameter**	**Greenfile**	**PubMed**	**Agricola**	**ProQuest**	**Science Direct**	**Web of Science**	**TOTAL HITS***
biofuels<br>*OR*<br>ethanol, corn-grain ethanol, bioenergy, crop-based biofuels, biodiesel, soybean biodiesel, biogas, algal biofuels, cellulosic bioenergy, bioenergy feed stock	*AND*	food security<br>*OR*<br>food prices, food availability, food insecurity, food production	595	537	522	661	810	3292	6417
